# miR-15a-5p outperforms anti-VEGF drug in ocular neovascularization by providing dual anti-angiogenic and neuroprotective effects

**DOI:** 10.7150/thno.134644

**Published:** 2026-07-29

**Authors:** Hui Zhang, Xinyue Yu, Fuhua Yang, Rongguo Yu, Liangzhang Tan, Jinying An, Huan Wang, Yiran Cui, Wenrui Linghu, Yue Wang, Jiahui Wu, Xiaomin Zhang, Xiaorong Li

**Affiliations:** 1 Tianjin Key Laboratory of Retinal Functions and Diseases, Tianjin Branch of National Clinical Research Center for Ocular Disease, Eye Institute and School of Optometry, Tianjin Medical University Eye Hospital, Tianjin, China.; 2 Tianjin Eye Hospital, Tianjin Key Laboratory of Ophthalmology and Visual Science, Tianjin Eye Institute, Nankai University Affiliated Tianjin Eye Hospital; Clinical College of Ophthalmology, Tianjin Medical University; Tianjin, China.

**Keywords:** miR-15a-5p, ocular neovascularization, retinal neuroprotection, endothelial-to-mesenchymal transition (EndoMT), anti-VEGF therapy

## Abstract

**Background:**

Pathological ocular neovascularization is a major driver of vision-threatening retinal diseases. This study aimed to investigate the role and therapeutic potential of miR-15a-5p in ocular neovascular disorders.

**Methods:**

miR-15a-5p expression levels were assessed in intraocular fluids from patients with ocular neovascular diseases. Functional assays were performed in retinal endothelial cells under pathological conditions to evaluate proliferation and endothelial-to-mesenchymal transition. *In vivo*, miR-15a-5p was delivered via intravitreal injection in oxygen-induced retinopathy (OIR) and laser-induced choroidal neovascularization (CNV) mouse models. Therapeutic effects on pathological neovascularization were analyzed and compared with anti-VEGF treatment, including assessments of retinal structural integrity, retinal function, gliosis, and fibrotic changes. miR-15a-5p–knockout mice were used to examine retinal vascular developmental abnormalities and enhanced neovascular responses following miR-15a-5p deficiency. Safety evaluations of systemic and ocular administration were performed in both healthy and neovascularized mice. Mechanistic studies investigated whether miR-15a-5p directly targeted VEGF and Smad2.

**Results:**

miR-15a-5p was significantly upregulated in intraocular fluids from patients with ocular neovascular diseases. Overexpression of miR-15a-5p inhibited retinal endothelial cell proliferation and endothelial-to-mesenchymal transition *in vitro*. In OIR and CNV models, miR-15a-5p treatment reduced retinal neovascularization, decreased reactive gliosis, and maintained retinal thickness and electrophysiological function. In miR-15a-5p–knockout mice, loss of miR-15a-5p impaired normal retinal vascular development. Mechanistically, miR-15a-5p directly targeted VEGF and Smad2, modulating angiogenic and fibrotic pathways. Compared with anti-VEGF therapy, miR-15a-5p demonstrated stronger anti-fibrotic and neuroprotective effects without affecting postnatal development or systemic metabolism. No ocular or systemic toxicity was observed at therapeutic doses.

**Conclusions:**

miR-15a-5p regulates angiogenesis and fibrosis by targeting VEGF and Smad2. These findings suggest that miR-15a-5p is a promising therapeutic candidate for the treatment of ocular neovascular diseases.

## Introduction

Pathological blood vessel growth contributes to several vision-threatening disorders, such as wet age-related macular degeneration (wAMD) and proliferative diabetic retinopathy (PDR). Vascular endothelial growth factor (VEGF) is widely recognized as a critical regulator of retinal neovascularization 1-3. Although anti-VEGF therapies provide therapeutic benefits, repeated injections may increase the risks of retinal neurodegeneration and fibrosis 4-6. Accordingly, to address the limitations of anti-VEGF therapy for neovascular ocular diseases, new therapeutic approaches are desperately needed.

As intrinsic regulators of gene expression, miRNAs regulate several biological processes, such as angiogenesis, fibrosis, and immunomodulation. Increasing evidence has shown that cells secrete different subtypes of extracellular vesicles (EVs), including exosomes, microvesicles, and apoptotic bodies, all of which can carry functional miRNAs and participate in intercellular communication 7. According to the MISEV2023 guidelines, EVs should be operationally defined based on their physical characteristics after isolation; therefore, vesicles with diameters smaller than 200 nm are termed small EVs (sEVs), whereas larger vesicles are categorized as medium/large EVs (m/lEVs) 8. Our previous study revealed differential expression of plasma-derived sEV miRNAs during the development and progression of PDR 9. Among them, miR-15a-5p was one of the markedly upregulated miRNAs.

Accumulating observations demonstrate that miR-15a-5p plays a crucial role in modulating diverse biological processes related to DR, including leukocyte adhesion, cell-cycle regulation, and apoptosis 10. Previous findings suggest that miR-15a-5p suppresses retinal inflammation by reducing inflammatory cytokine secretion and leukocyte infiltration 11. Another study reported that decreased miR-15a-5p expression directly influenced ceramide activation in the retina, resulting in a persistent low-grade chronic inflammatory response 12. Despite these findings, the role of miR-15a-5p in pathological ocular neovascularization has not been fully characterized.

In this study, increased expression of miR-15a-5p was observed in circulating sEVs and vitreous samples from PDR patients and in aqueous humor from wAMD patients. We next examined its function in ocular angiogenesis. Our findings suggest that miR-15a-5p exerts anti-angiogenic effects comparable to anti-VEGF drugs, but confers additional benefits, including vascular normalization, retinal neuroprotection, and enhanced systemic safety. Mechanistically, we identified VEGF signaling and Smad2 signaling as the downstream pathways modulated by miR-15a-5p. Collectively, miR-15a-5p represents a promising therapeutic candidate for neovascular eye diseases.

## Materials and Methods

### Study design

The Ethics Committee of Tianjin Medical University Eye Hospital accepted this study (Approval No. 2017KY-01). Every participant provided written informed consent. The study population consisted of nondiabetic healthy controls and patients with type 2 diabetes recruited from the outpatient department between December 2020 and December 2021. Participants were enrolled based on the following eligibility criteria: (1) age of 40–81 years, (2) diagnosis of type 2 diabetes, and (3) willingness to participate. Exclusion was determined according to the following criteria: (1) ocular vascular diseases unrelated to diabetes, (2) systemic or ocular infectious diseases, (3) history of intraocular surgery, (4) history of cerebral infarction or myocardial infarction within the past year, (5) history of retinal laser therapy within the past year, (6) grade III hypertension, and (7) malignancy.

### Sample collection and storage

The preparation of plasma samples followed established protocols 13. Vitreous specimens were obtained from subjects who underwent pars plana vitrectomy. During the procedure, approximately 400 μL of vitreous fluid was aspirated using a 27-gauge vitrectomy cutter. Samples were processed and stored as described in earlier studies 13.

### sEV isolation

All centrifugation procedures, including ultracentrifugation, were carried out at 4 °C. To reduce viscosity, 1 mL of plasma from each participant was diluted in phosphate-buffered saline (PBS) (Gibco, USA). The diluted plasma was subjected to a series of centrifugation steps, as previously described 13. The clarified supernatant was ultracentrifuged in an SW41 Ti swing rotor at 110,000 × g for 2 hours to pellet the sEVs. We further purified the sEVs by resuspending the pellet in 11.5 mL PBS. We performed a final ultracentrifugation (150,000 × g, 120 min). The sEV pellet was then kept at -80 °C after being resuspended in 100 μL of Buffer XE (Solarbio, Beijing, China) or PBS.

### Coomassie Brilliant Blue (CBB) staining

A total of 12 μg of denatured protein was loaded onto a 10% polyacrylamide gel, followed by electrophoretic separation at 100 V. After electrophoresis, the gel was carefully removed and incubated in CBB staining solution with gentle agitation at 25 ℃ for 2 h. The gel was washed until the background became clear, and images were captured.

### Nanoparticle tracking analysis (NTA)

Each sample was detected on a NanoSight NS300 system (Malvern Panalytical, UK). The temperature was set to 25 ℃, with a blue 488 nm laser, a flow rate of 50, and the detection mode set to automatic. For data acquisition, each sample was subjected to three consecutive injections, and the mean peak value was calculated as the representative measure.

### Transmission electron microscope (TEM) evaluation

We performed TEM sample preparation by resuspending the sEVs in 50 μL of PBS and depositing them onto a copper grid. The grid was then negatively stained with 30 μL of 1% phosphotungstic acid for 4 min and examined under a Hitachi 7700 TEM (Hitachi 7700, Japan).

### Cell Culture

HRMECs were purchased from Angio-Proteomie (Boston, USA). Cell cultivation followed established protocols 13. ARPE-19 cells were purchased from the American Type Culture Collection (ATCC, Manassas, VA, USA). The ARPE-19 cell line was cultured in complete DF12 medium. Complete DF12 medium contained fetal bovine serum (10%) and penicillin-streptomycin (1%). Tests confirmed the absence of mycoplasma contamination.

### Cell transfection

The miRNA mimics and inhibitors targeting hsa-miR-15a-5p were generated by GenePharma (Suzhou, China). The oligonucleotide sequences used were as follows: hsa-miR-15a-5p mimic sense: 5′-UAGCAGCACAUAAUGGUUUGUG-3′; antisense: 5′-CACAAACCAUUAUGUGCUGCUA-3′; hsa-miR-15a-5p inhibitor: 5′-CACAAACCAUUAUGUGCUGCUA-3′. Cells were transfected with Lipofectamine 3000.

### Immunofluorescence cell staining

The immunofluorescence assay in cells followed established protocols 13. The antibodies were as follows: vimentin (1:300, Abcam, ab92547), α-SMA (1:500, Abcam, ab124964), and CD31 (1:150, Abcam, ab9498).

### Western blot (WB)

This assay was carried out as previously described 3. Proteins from plasma, sEVs, cultured cells, and retinal tissues were extracted using RIPA buffer. Protein samples containing the same total amount of protein were resolved by SDS–PAGE and subsequently transferred to PVDF membranes. The membranes were then blocked and incubated with the indicated primary antibodies at 4 °C overnight. After washing, the membranes were incubated with secondary antibodies at room temperature. All washing steps were performed using TBST (Solarbio, Beijing, China). A list of the primary antibodies used in this study is provided in Table S4.

### RNA extraction and quantitative RT-PCR

Total RNA was extracted utilizing the Universal RNA Purification Kit (EZBioscience, China) and from plasma and plasma-derived sEVs using the exoEasy Maxi Kit (Qiagen, Germany). The RevertAid Kit (Thermo Fisher, USA) was used to convert mRNA to cDNA. The miScript Reverse Transcription Kit (Qiagen) was used to create cDNA for miRNA. Real-time quantitative PCR was carried out using a LightCycler 480 (Roche, Switzerland).

Primer sequences are listed below. U6: forward 5′-CTCGCTTCGGCAGCACA-3′; reverse 5′-AACGCTTCACGAATTTGCGT-3′. cel-miR-39: forward 5′-ACACTCCAGCTGGGTCACCGGGTGTAAATC-3′; reverse 5′-CTCAACTGGTGTCGTGGAGTCGGCAATTCAGTTGAGCAAGCTGA-3′. GAPDH: forward 5′-ATGGAAATCCCATCACCATCTT-3′; reverse 5′-CGCCCCACTTGATTTTGG-3′. Smad2: forward 5′-TGTTTTCAGTTCCGCCTCCA-3′; reverse 5′-GCGTGAATGGCAAGATGGAC-3′. hsa-miR-15a-5p: forward 5′-CGGGCTAGCAGCACATAATGG-3′. TNF-α: forward 5′-GCCTCTTCTCATTCCTGCTT-3′; reverse 5′-CTCCTCCACTTGGTGGTTTG-3′; VEGF: forward 5′- CCCGACAGGGAAGACAAT-3′; reverse 5′- TCTGGAAGTGAGCCAACG-3′; ICAM-1: forward 5′- CTGGGCTTGGAGACTCAGTG-3′; reverse 5′-CCACACTCTCCGGAAACGAA-3′.

### Cell viability assay

HRMECs were trypsinized and seeded into 96-well plates. After 24 hours of stimulation, the original culture medium was removed, and CCK-8 reagent (MCE, China) was added. Absorbance was recorded using a Tecan microplate reader (Tecan, Switzerland).

### Scratch assay

Twenty-four hours after the transfection, a straight and uniform scratch was created on the adherent cells using a pipette tip. Scratch images were obtained at both 0 h and 18 h. The corresponding scratch area was subsequently determined by quantification with ImageJ software (v1.52, USA).

### Transwell cell migration assay

To evaluate the migratory capacity of hsa-miR-15a-5p-transfected cells, a Transwell migration assay was employed. After pretreatment, HRMECs were seeded at 8,000 cells per well in the upper chambers of Transwell inserts (Corning, New York, USA). After 1 day, the chambers were fixed with PFA and stained with crystal violet. Cells were counted under a microscope. Counts were obtained from five randomly selected microscopic fields.

### Tube formation assay

This assay was carried out based on established protocols 13. After pretreatment, the HRMECs were seeded onto Matrigel-coated 48-well plates (Corning, Switzerland). A microscope (Olympus Corporation, Japan) was used to image five randomly selected fields in each well after 3 h. Quantification of the total capillary-like structure length was performed with ImageJ software (v1.52, USA).

### Dual-luciferase reporter gene experiment

The 3′UTRs of VEGF and Smad2 were separately cloned into the pmirGLO dual-luciferase reporter vector (GenePharma, China). To assess the effect, reporter plasmids were introduced into 293T cells via Lipofectamine 3000. Reporter gene activity was evaluated with a Dual-Luciferase® Reporter Assay System (GenePharma, China) as described in the supplied protocol.

### Animals

All animal procedures were performed in compliance with ARVO animal research guidelines. The study protocol received approval from the Animal Ethics and Welfare Committee of Tianjin Medical University Eye Hospital (Approval No. TJYY2019091124). Housing of the mice was provided in a standard laboratory environment (23 ± 1.5 °C, 42 ± 8% relative humidity). Male miR-15a/16^-/-^ mice (miR-15a-5p KO mice) and their WT littermates were obtained from GemPharmatech Co. Ltd. (China) for subsequent experiments.

### Oxygen-induced retinopathy (OIR)

An OIR model was performed with C57BL/6J mice by exposing newborn pups. They were exposed to 75% oxygen from P7 to P12 together with their nursing dams. The nursing dams were replaced every 24 h. At P12, the OIR mice underwent intravitreal injection.

### Retinal vaso-obliteration and neovascularization quantification

OIR mice that received intravitreal injections were sacrificed at P17. Retinas were dissected, fixed in 4% PFA, and cut into four radial quadrants. Retinas were stained with IB4 (Thermo Fisher, USA) overnight at 4 °C. Following the wash steps, the samples were prepared for imaging by mounting them with fluorescence mounting medium (Vector, Germany) under coverslips. Areas of neovascularization (NV) and vasoobliteration (VO) were quantified by a blinded investigator using Adobe Photoshop software (Adobe Systems, USA).

### Laser-induced choroidal neovascularization

After anesthetizing seven-week-old mice with intraperitoneal tribromoethanol (300 mg/kg), laser photocoagulation was performed using a laser system (Phoenix, USA). Immediately after photocoagulation, agomir-15a-5p, agomir-NC, or the anti-VEGF drug were administered intravitreally.

### Intravitreal injection

Animal procedures were performed under anesthesia with sterile Avertin (tribromoethanol, 300 mg/kg, i.p.). Subsequent to pupil dilation using 0.5% tropicamide, a Hamilton microsyringe (34-gauge, USA) was employed for a single intravitreal administration per mouse. The injected solution (1 µL) consisted of PBS (serving as negative control), agomir, or anti-VEGF antibody, with meticulous technique to prevent lens injury. The anti-VEGF drug used in this study was ranibizumab. Ranibizumab dosing in mice was determined based on vitreous volume conversion between human and mouse eyes. The average vitreous volume in the adult human eye is approximately 4 mL, whereas that in the mouse eye is 5 μL. In clinical practice, the standard intravitreal dose of ranibizumab in humans is 0.5 mg per injection. Based on vitreous volume normalization, the theoretical equivalent dose in mice was calculated to be approximately 0.5 μg per eye. Considering that intraocular drug clearance is faster in mice than in humans and that partial reflux may occur during intravitreal injection in mice, we selected 1–10 μg/eye as the dose range for therapeutic evaluation based on previous literature reports and preliminary experiments. Dose-dependent therapeutic effects were further assessed in the OIR mouse model.

### Enzyme-linked immunosorbent assay

Using commercial VEGF ELISA kits (Abcam, USA), samples of cell culture medium were analyzed according to the protocol. TNF-α levels were measured using a commercial kit (Abcam, USA). Briefly, retinal lysates were prepared from mouse retinas, and 100 μL of each sample was processed following the recommended procedure.

### Electroretinogram

Electroretinography was performed as previously described 3. Electroretinogram recordings were conducted on mice after a 24-hour dark adaptation period, utilizing the Phoenix Research Labs system (USA) under dark conditions. For the recordings, corneal contact was established with gold electrodes. Recordings were obtained after each flash. The interstimulus intervals were set at 1 s (0.1 log), 10 s (1 log), and 30 s (2.2 log).

### Optical coherence tomography (OCT)

Following anesthesia and pupillary dilation, retinal imaging was conducted using an OCT system (Spectralis, Heidelberg, Germany) to assess structural alterations. For quantitative analysis, a peripapillary circular scan (ETDRS) was employed, and the resulting measurements were reported as mean retinal thickness in micrometers.

### Immunofluorescent staining

Eyeballs were dissected, immediately embedded in O.C.T. compound (SAKURA, USA), and frozen. Retinal sections (8 μm thick) were prepared. Fixation, blocking, and staining were carried out as reported in earlier studies 3. Following mounting with a fluorescence mounting medium (Vector, Burlingame, CA, USA), coverslips were applied to the samples. Information on all primary antibodies employed in this study is summarized in Table S4.

### TUNEL assay

The preparation of retinal sections was implemented following established protocols. Briefly, retinas were embedded in O.C.T., snap-frozen, and cut into 6 μm-thick sections. DNA fragmentation was examined by TUNEL staining using a commercial kit (Roche, Switzerland). Fluorescence images were obtained with an LSM800 confocal microscope (ZEISS, Germany).

### Hematoxylin and eosin (H&E) staining

Organs or eyes were removed, fixed, and then embedded in paraffin. Horizontal paraffin sections of each retina were prepared and stained with H&E. Microscopic image acquisition for each section was conducted using an Olympus light microscope (Tokyo, Japan).

### Detection of serum biochemical indicators

The manufacturer’s instructions for serum biochemical indicator detection were followed for all assay procedures (ElabScience, China). Briefly, a standard curve was established, and serum samples were diluted to the required concentration and dispensed into 96-well plates together with the corresponding working solutions. Measurements were taken on a microplate reader to determine absorbance; biochemical indicator levels were then derived from the standard curve.

### Statistical analysis

Data are presented as the mean ± SD. Statistical analyses and graphics were generated using GraphPad Prism (GraphPad, USA), SPSS version 23 (IBM, USA), and Adobe Photoshop (Adobe Systems). Data were analyzed as follows: an unpaired two-tailed Student's t-test was used for comparisons between two groups, and a one-way ANOVA or two-way ANOVA with a post-hoc test was used for comparisons among three or more groups. The rank-sum test was employed to analyze data with unequal variances or data that were not normally distributed. The threshold for statistical significance was established at P < 0.05. Sample size was determined by a priori power analysis (80% power; α = 0.05) using effect size and variance estimates obtained from preliminary data and published literature.

## Results

### miR-15a-5p is upregulated in biological samples from patients with PDR and wAMD

**Figure 1:**
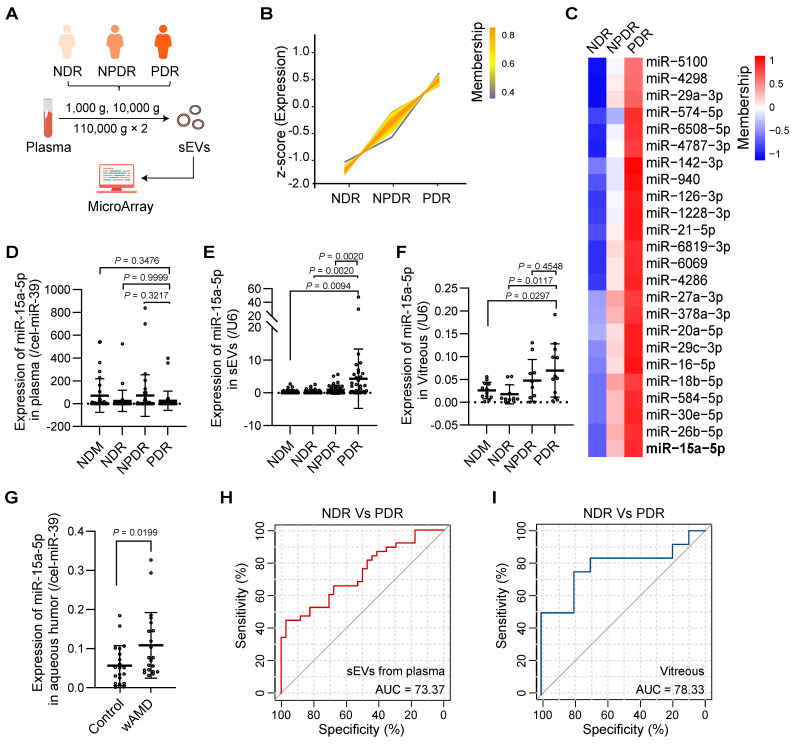
** Elevated miR-15a-5p levels in circulating sEVs and vitreous from patients with PDR and in the AH of patients with wAMD. A**. Flowchart of sEV microarray analysis. **B**. A miRNA cluster showing progressive upregulation across the disease continuum from NDR to NPDR and PDR. **C**. Heat map of upregulated miRNAs identified in the profiling analysis. **D**. Expression levels of miR-15a-5p in the plasma from the validation cohort across the NDM (*n* = 34), NDR (*n* = 34), NPDR (*n* = 36), and PDR (*n* = 38) groups. **E**. Expression levels of miR-15a-5p in plasma-derived sEVs from the validation cohort across the NDM (*n* = 34), NDR (*n* = 34), NPDR (*n* = 36), and PDR (*n* = 38) groups. **F**. Expression levels of miR-15a-5p in the vitreous humor from the validation cohort across the NDM (*n* = 12), NDR (*n* = 10), NPDR (*n* = 10), and PDR (*n* = 12) groups. The indications for vitrectomy in the NDM, NDR, and NPDR groups were macular holes or epiretinal membranes. **G**. Expression levels of miR-15a-5p in the AH from the validation cohort across the Control (*n* = 21) and wAMD (*n* = 21) groups. The control group comprised patients with cataracts. **H.** Receiver operating characteristic (ROC) curve analysis of sEV-derived miR-15a-5p for discriminating PDR from NDR. **I.** ROC curve analysis of vitreous miR-15a-5p for discriminating PDR from NDR. Data in** D, E, F,** and** G** are presented as mean ± SD and analyzed using one-way ANOVA (**D, E, F**) or a two-tailed unpaired Student’s t-test (**G**).

Our previously published miRNA sequencing data from plasma-derived sEVs of patients with no diabetic retinopathy (NDR), non-proliferative diabetic retinopathy (NPDR), and PDR revealed a cluster of miRNAs that increased progressively with disease severity (**Figure 1A–B**) 9. Among them, miR-15a-5p was highly abundant and markedly upregulated (**Figure 1C**). To validate this finding, we analyzed an independent cohort comprising nondiabetic individuals (NDM), NDR, NPDR, and PDR patients. Participant characteristics are presented in **Table S1**. Plasma was collected from these subjects and quantitative PCR revealed no marked differences in miR-15a-5p expression across the groups (**Figure 1D**). Plasma-derived sEVs were isolated and characterized, exhibiting typical morphological and molecular characteristics of sEVs (**Figure S1A–D**). Consistent with the sequencing results, miR-15a-5p levels in plasma-derived sEVs were notably upregulated in patients with PDR in contrast to the other groups (**Figure 1E**). Next, we examined vitreous samples from another independent cohort of patients with NDM, NDR, NPDR, and PDR (**Table S2**). Similarly, vitreous samples showed higher miR-15a-5p levels in patients with PDR than in NDM and NDR controls (**Figure 1F**). We also observed elevated expression of miR-15a-5p in the aqueous humor (AH) of patients with wAMD (**Table S3, Figure 1G**). Furthermore, the receiver operating characteristic (ROC) curve analysis revealed that both vitreous miR-15a-5p and sEV-derived miR-15a-5p effectively distinguished PDR from NDR (**Figure 1H–I**). Together, these findings demonstrate elevated miR-15a-5p levels in patients with PDR and wAMD, suggesting its potential involvement in ocular neovascular diseases.

### miR-15a-5p suppresses the proliferation of HRMECs

**Figure 2:**
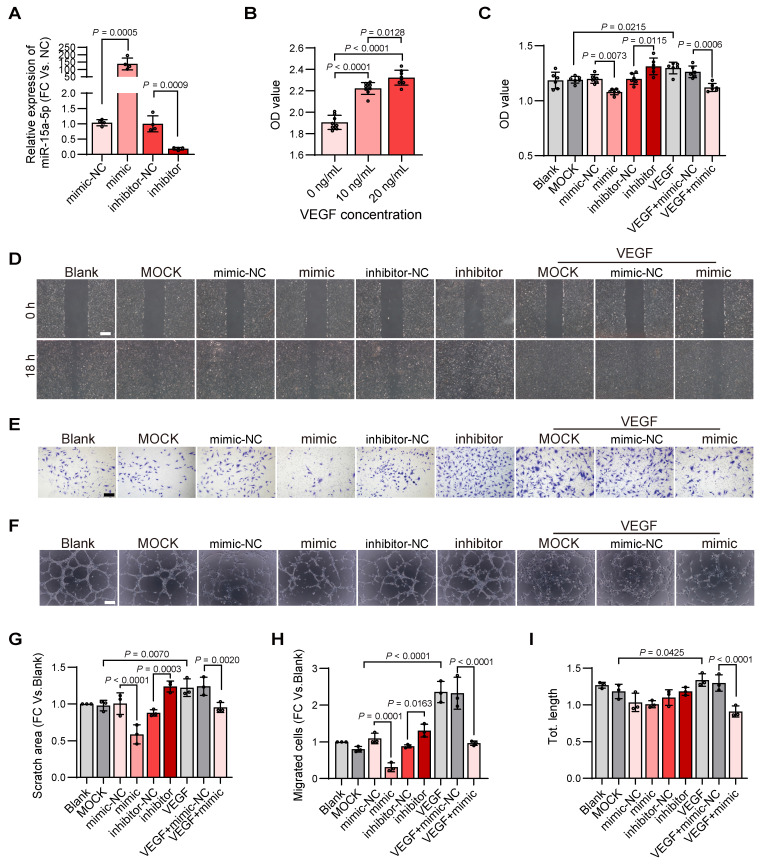
** miR-15a-5p regulates HRMEC proliferation and migration. A**. Transfection efficiency of miR-15a-5p mimics and inhibitors in HRMECs (*n* = 4). **B**. CCK-8 assay showing VEGF concentrations stimulating HRMEC proliferation (*n* = 8). **C**. CCK-8 assay showing the proliferation of HRMECs subjected to different treatments, with or without VEGF stimulation (*n* = 6). **D**. Representative images of the wound-healing assay in HRMECs subjected to different treatments, with or without VEGF stimulation. Scale bar: 200 μm. **E**. Representative images of the transwell migration assay in HRMECs subjected to different treatments, with or without VEGF stimulation. Scale bar: 100 μm. **F**. Representative images of the HRMEC tube formation assay subjected to different treatments, with or without VEGF stimulation. Scale bar: 100 μm. **G**. Quantification of wound closure in the wound-healing assay (*n* = 3). **H**. Quantification of migrated cells in the transwell assay (*n* = 3). **I**. Quantification of total tube length in HRMECs under different treatment conditions (*n* = 3). Treatments include: MOCK (transfection reagent only), mimic-NC (transfected with non-targeting mimic control), mimics (transfected with miR-15a-5p mimics), inhibitor-NC (transfected with non-targeting inhibitor control), and inhibitor (transfected with miR-15a-5p inhibitor). Data in **A, B, C, G, H,** and** I** are presented as mean ± SD and were compared using one-way ANOVA.

As retinal neovascularization represents a central pathological event in PDR and is largely driven by endothelial activation, we examined the regulatory role of miR-15a-5p in HRMECs. We confirmed that transfection with miR-15a-5p mimics led to an approximately 150-fold increase in its expression, whereas transfection with inhibitors reduced its expression by approximately 80% (**Figure 2A**). Prior to functional assays, we determined the optimal VEGF concentration required to induce aberrant proliferation of HRMECs *in vitro* (**Figure 2B**). The effects of miR-15a-5p on HRMEC angiogenic function were evaluated by measuring cell growth, motility, and capillary-like network formation after modulation of miR-15a-5p expression with mimics or inhibitors. Enhanced expression of miR-15a-5p markedly suppressed HRMEC proliferation (**Figure 2C**), wound closure (**Figure 2D, G**), transwell migration (**Figure 2E, H**), and attenuated VEGF-induced enhancement of angiogenic activity (**Figure 2F, I**). Conversely, suppression of miR-15a-5p led to enhanced endothelial proliferation and migration.

### Loss of miR-15a-5p compromised retinal vascular development in the superficial and deep vascular plexuses

To investigate the role of miR-15a-5p in retinal vascular development and neovascularization, we obtained miR-15a-5p knockout (KO) mice from GemPharmatech. Compared to their wild-type (WT) littermates, KO mice showed no abnormalities in body weight or overall physical development before weaning (1 week old) or in adulthood (8 weeks old) (**Figure S2A–B**). Retinal sections from KO mice exhibited normal morphology without obvious histological abnormalities (**Figure S2C**). Optical coherence tomography (OCT) analysis showed that the retinal thickness increased in KO mice (**Figure S2D**), particularly within the inner plexiform layer (**Figure S2E–F**). Functionally, electroretinography (ERG) under a 1.0-log cd·s/m² flash stimulus showed reduced amplitudes of both the a- and b-waves in KO mice compared with those in WT controls (**Figure S2G–H**), indicating mild impairment of retinal function associated with miR-15a-5p deficiency. In addition, we performed quantitative analysis of retinal vascular morphology in adult KO mice and observed a significant reduction in the number of vascular junctions, total vessel length, and mesh number compared with WT controls, indicating impaired retinal vascular complexity in KO mice (**Figure S2I–M**).

Next, we examined the effect of miR-15a-5p deficiency on retinal vascular development. The retinal vasculature was visualized using isolectin B4 (IB4) staining on postnatal day 7 (P7). In WT mice, the superficial vascular plexus extended to the retinal periphery, whereas vascular outgrowth was markedly delayed in KO mice (**Figure S3A**). Quantitative analysis revealed that the retinas of KO mice exhibited significantly reduced superficial vascular coverage (**Figure S3B**), a lower number of vascular junctions (**Figure S3C**), and shorter total vessel length and branch length (**Figure S3D–E**). During retinal development, astrocytes provide scaffolds for vessel growth. Astrocytes were largely absent in the peripheral retinas of KO mice (**Figure S3F**). In the mid-retinal region, the vascular network displayed enlarged mesh-like structures, with some astrocytes failing to associate closely with the blood vessels (**Figure S3G–I**). These results indicate that the loss of miR-15a-5p disrupts astrocyte patterning and consequently impairs retinal vascular patterning. In addition, we examined the formation of the superficial and deep retinal vasculature at P9 (**Figure S4A**). KO mice displayed reduced superficial vascular complexity and impaired deep vascular development (**Figure S4B–D**), further indicating that miR-15a-5p deficiency delayed both superficial and deep normal retinal vascular development.

### Intravitreal injection of miR-15a-5p inhibited pathological ocular neovascularization

**Figure 3:**
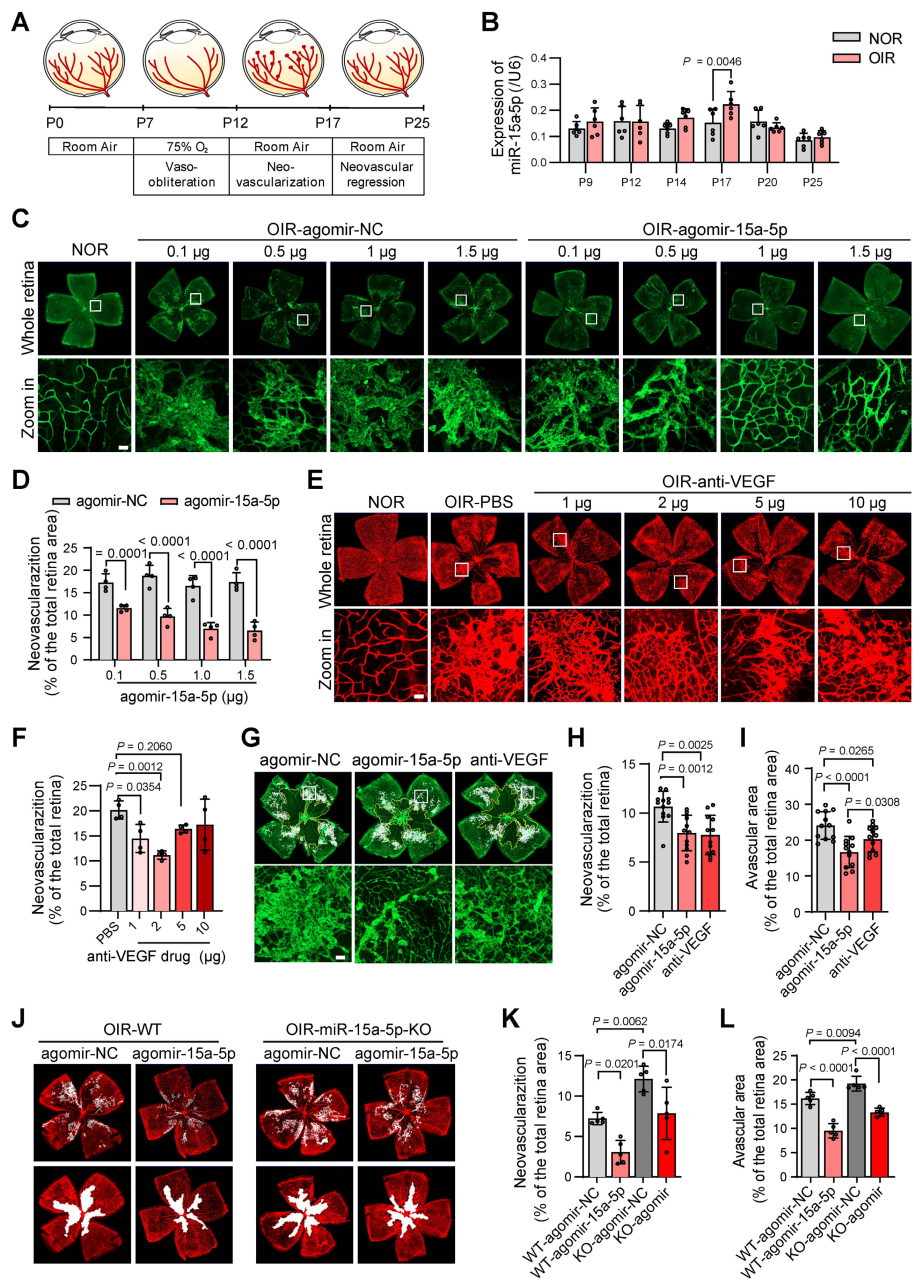
** miR-15a-5p administration suppresses pathological retinal neovascularization in the OIR model. A**. Schematic of the OIR model. **B**. Quantitative RT-PCR analysis of miR-15a-5p levels in the retinas of OIR mice (*n* = 6). **C**. Representative retinal flat-mounted images illustrating the dose-dependent inhibitory effects of agomir-15a-5p on pathological neovascularization compared to the negative control agomir (agomir-NC). Scale bar: 20 μm. **D**. Quantification of pathological retinal neovascularization in the retinas (*n* = 4). **E**. Representative retinal flat-mounted images illustrating the dose-dependent inhibitory effects of anti-VEGF drug on pathological neovascularization. Scale bar: 20 μm. **F**. Quantification of pathological retinal neovascularization (*n* = 4). **G**. Representative retinal flat-mount images illustrating the therapeutic effects of agomir-15a-5p and anti-VEGF treatment on pathological neovascularization (white area) and retinal nonperfusion areas (yellow area). Scale bar: 20 μm. **H**. Quantification of pathological retinal neovascularization in the agomir-15a-5p- and anti-VEGF-treated groups compared with controls (*n* = 12). **I**. Quantification of retinal nonperfusion areas in agomir-15a-5p- and anti-VEGF-treated groups versus controls (*n* = 12). **J**. Representative retinal flat-mount images illustrating pathological neovascularization (upper panels, white) and nonperfusion areas (lower panels, white) in miR-15a-5p KO mice versus WT mice. **K**. Quantification of pathological retinal neovascularization in miR-15a-5p KO mice versus WT mice (*n* = 5). **L**. Quantification of retinal nonperfusion areas in miR-15a-5p KO mice versus WT mice (*n* = 5). Data in **B, D, F, H, I, K,** and** L** are presented as mean ± SD and analyzed using one-way ANOVA.

Oxygen-induced retinopathy (OIR) and choroidal neovascularization (CNV) mouse models were established to determine the anti-angiogenic efficacy of miR-15a-5p. In the OIR model, miR-15a-5p expression was mildly upregulated (1.4-fold) at P17 compared to that in normal retinas (**Figure 3A–B**). Intravitreal administration of agomir-15a-5p was subsequently performed at P12, and pharmacokinetic profiling showed an approximately 3-fold increase in retinal miR-15a-5p levels within 24–48 hours (**Figure S5A–C**). Next, dose–response analyses in OIR mice revealed a dose-dependent reduction in retinal neovascularization following intravitreal injection of agomir-15a-5p at P12 (**Figure 3C–D**). For comparison, the optimal *in vivo* inhibitory dose of the anti-VEGF drug (ranibizumab) was determined (**Figure 3E–F**). Based on these results, doses of 1 μg of agomir-15a-5p and 2 μg of anti-VEGF drug, both near their respective maximal effective doses, were selected for subsequent experiments. Both treatments significantly suppressed pathological retinal neovascularization and produced comparable therapeutic effects (**Figure 3G–H**). However, agomir-15a-5p induced a significantly greater reduction in the central avascular area than the anti-VEGF drug (**Figure 3I**), suggesting its role in promoting vascular remodeling. Consistently, miR-15a-5p KO mice displayed markedly increased retinal neovascularization and larger avascular areas compared to WT controls, which were effectively reversed by the intravitreal injection of agomir-15a-5p (**Figure 3J–L**).

Furthermore, in the laser-induced CNV model, agomir-15a-5p exhibited efficacy comparable to that of the anti-VEGF drug in reducing CNV leakage and neovascular cluster area, consistent with the findings in OIR mice (**Figure S6A–F**). Notably, KO mice developed significantly larger CNV lesions than WT controls, indicating that the loss of miR-15a-5p exacerbates pathological angiogenesis. Intravitreal administration of agomir-15a-5p effectively reversed this phenotype (**Figure S6G–H**). Thus, while both agomir-15a-5p and the anti-VEGF drug efficiently inhibited pathological neovascularization, agomir-15a-5p additionally facilitated vascular remodeling within non-perfused retinal regions in the OIR model.

### miR-15a-5p exhibits superior neuroprotective effects compared with anti-VEGF therapy in OIR and CNV models

**Figure 4:**
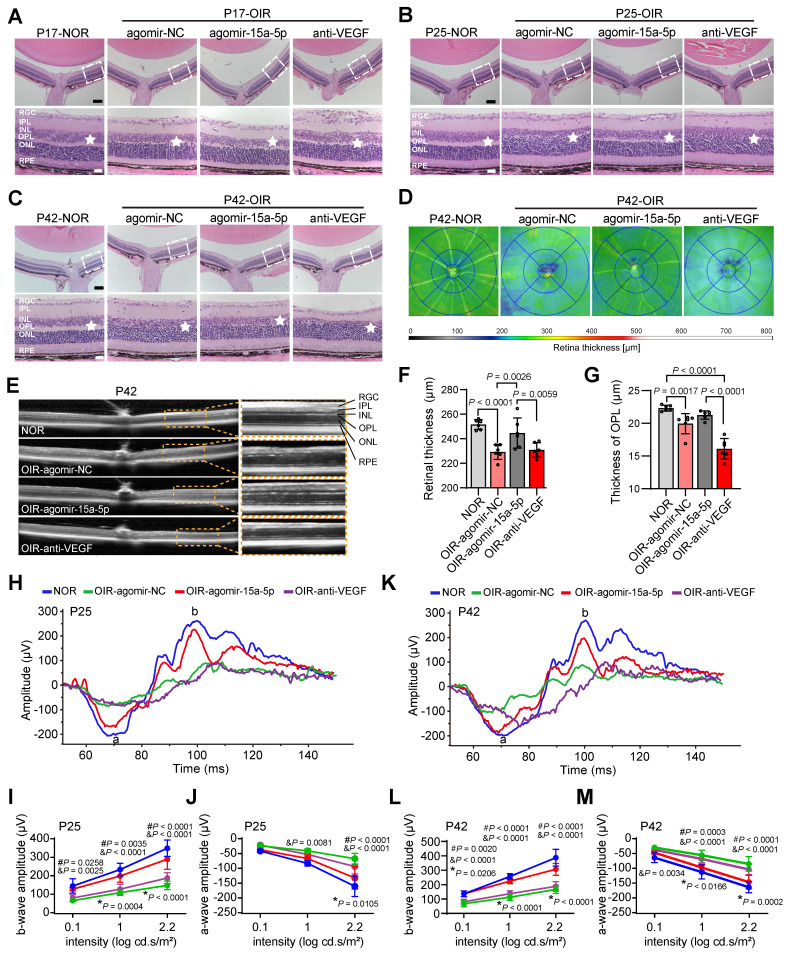
** Intravitreal injection of miR-15a-5p improved retinal structure and visual function in OIR mice. A-C**. H&E–stained retinal sections from OIR and NOR mice at P17 (**A**), P25 (**B**), and P42 (**C**). Pentagrams indicate the OPL. Scale bar: 100 μm (upper panel), 20 μm (bottom panel). **D**. Representative OCT topographic maps of retinal thickness in OIR and NOR mice at P42. **E**. Representative OCT cross-sections showing retinal architecture in OIR and NOR mice at P42. **F**. Quantitative analysis of retinal thickness (*n* = 6). **G**. Quantitative analysis of OPL thickness (*n* = 6). **H**. Representative ERG waveforms of OIR and NOR mice at P25 in response to a 1.0 log cd·s/m² light flash. **I-J**. Quantitative analysis of b-wave (**I**) and a-wave (**J**) amplitudes in OIR and NOR mice at P25 under different light intensities (*n* = 4–6). **K**. Representative ERG waveforms of OIR and NOR mice at P42 in response to a 1.0 log cd·s/m² light flash. **L-M**. Quantitative analysis of b-wave (**L**) and a-wave (**M**) amplitudes in OIR and NOR mice at P42 under different light intensities (*n* = 5–6). Data in **F, G, I, J, L,** and **M** are presented as mean ± SD and were compared using one-way ANOVA (**F, G**) and two-way ANOVA (**I, J, L, M**). & denotes a significant difference between agomir-NC and NOR; # denotes a significant difference between agomir-15a-5p and agomir-NC; * denotes a significant difference between agomir-15a-5p and anti-VEGF.

Histological examination from P17 to P42 revealed progressive structural deterioration of OIR retinas, with distinct temporal patterns between treatment groups (**Figure 4A–C**). At P17, retinas from anti-VEGF-treated mice exhibited thinning of the outer plexiform layer (OPL) (**Figure 4A**). By P25, OIR mice in the agomir-NC group showed evident OPL thinning, whereas those in the anti-VEGF group displayed a complete loss of OPL (**Figure 4B**). At P42, the anti-VEGF group exhibited the disappearance of the OPL and thinning of the ONL (**Figure 4C**). In contrast, the retinas treated with agomir-15a-5p maintained a well-preserved OPL structure throughout the observation period. Consistent with the histological observations, OCT analysis at P42 confirmed that agomir-15a-5p alleviated retinal thinning in OIR mice, whereas the anti-VEGF drug had no detectable effect (**Figure 4D–G**). To further evaluate retinal function, ERG was performed at both early (P25) and late (P42) time points after intravitreal injection. In OIR mice, both a- and b-wave amplitudes were markedly decreased relative to those in normal controls (**Figure 4H–M**). Agomir-15a-5p significantly improved these amplitudes at both time points, suggesting sustained functional recovery, whereas the anti-VEGF drug did not significantly alter the ERG response (**Figure 4H–M**).

In the CNV model, H&E staining showed that both agomir-15a-5p and the anti-VEGF drug promoted restoration of retinal structure at the lesion site, consistent with lesion regression (**Figure S7A**). Furthermore, ERG analysis revealed that only agomir-15a-5p significantly enhanced the a-wave and b-wave amplitudes, whereas the anti-VEGF drug showed no significant functional effect (**Figure S7B–D**). Together, these results indicated that although both agomir-15a-5p and the anti-VEGF drug effectively inhibited pathological neovascularization, only agomir-15a-5p provided additional benefits by promoting functional improvement.

### miR-15a-5p promotes vascular remodeling by preserving astrocytes and suppressing inflammation

**Figure 5:**
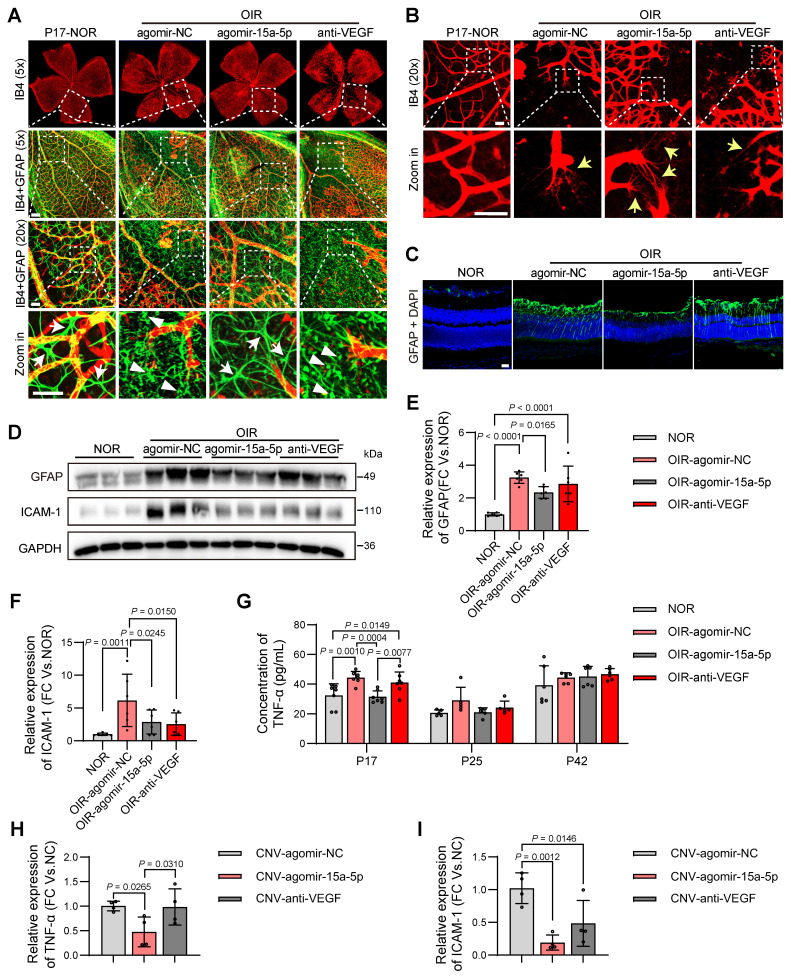
** miR-15a-5p promoted vascular remodeling and suppressed retinal inflammation in OIR and CNV models. A**. Representative images of non-perfused area margins in OIR and NOR retinas showing differences in astrocyte survival (white arrow) and Müller glia activation (white triangle). Scale bar: 80 μm (upper panel), 20 μm (middle panel and lower panels). **B**. Representative images of retinal endothelial tip cells and filopodia (yellow arrow) in OIR and NOR retinas. Scale bar: 20 μm. **C**. Representative immunofluorescence images of GFAP in retinal sections. Scale bar: 20 μm. **D**. Representative blots of GFAP and ICAM-1 in each group (GAPDH as the loading control). **E**. Quantification of GFAP protein levels normalized to GAPDH (*n* = 6). **F**. Quantification of protein ICAM-1 levels normalized to GAPDH (*n* = 6). **G**. ELISA quantification of TNF-α in retinal lysates at P17, P25, and P42 (*n* = 7, *n* = 5, *n* = 6). **H–I**. Quantitative RT-PCR analysis of TNF-α (**H**) and ICAM-1 (**I**) mRNA expression in retinal and choroidal tissues from CNV mice. Data in **E, F, G, H,** and** I** are presented as mean ± SD and analyzed using one-way ANOVA. The original Western blot images used for quantification in this study have been deposited in figshare and can be accessed at DOI: 10.6084/m9.figshare.30820226.

Attachment between endothelial tip cells and astrocytes is crucial for vascular remodeling at the margins of non-perfused retinal regions. In OIR mice, astrocytes within these avascular areas exhibited marked degeneration and enhanced GFAP reactivity in Müller glia at P17, whereas agomir-15a-5p treatment preserved a continuous astrocytic network with a typical star-shaped morphology (**Figure 5A**). Correspondingly, agomir-15a-5p–treated retinas showed a higher density and more organized distribution of endothelial tip cells, extending filopodia toward the preserved astrocytes (**Figure 5A–B**). In contrast, miR-15a-5p KO retinas displayed aggravated astrocyte loss and reduced endothelial sprouting, both of which were rescued by agomir-15a-5p administration (**Figure S8A–B**). Besides its function in vascular remodeling, miR-15a-5p has been shown to mitigate retinal inflammation. In OIR mice, agomir-15a-5p markedly reduced Müller cell activation (**Figure 5C–E**). Both agomir-15a-5p and anti-VEGF drug reduced intercellular adhesion molecule-1 (ICAM-1) expression (**Figure 5F**). Furthermore, agomir-15a-5p markedly reduced TNF-α expression at P17, while anti-VEGF treatment had minimal effect (**Figure 5G**). Similarly, in the CNV model, agomir-15a-5p downregulated TNF-α and ICAM-1 expression more effectively than the anti-VEGF drug (**Figure 5H–I**). These results demonstrate that miR-15a-5p promotes vascular remodeling in non-perfused retinal regions by maintaining astrocyte integrity and concurrently alleviating retinal inflammation, thus achieving broader protective effects than those of anti-VEGF therapy.

### miR-15a-5p suppresses VEGF expression and sustains ERK pathway inactivation

**Figure 6:**
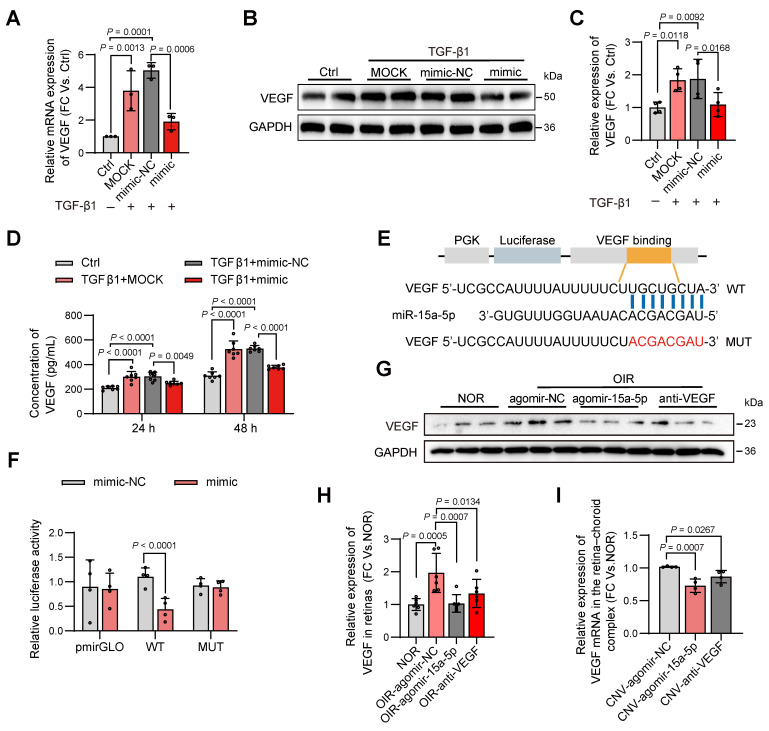
** miR-15a-5p directly regulated VEGF expression**. **A**. Quantitative PCR analysis showing that miR-15a-5p suppresses VEGF mRNA expression in RPE cells. **B-C**. Representative western blot images (**B**) and corresponding quantification demonstrating that miR-15a-5p reduces VEGF protein levels (**C**) in RPE cells (*n* = 4). **D**. ELISA analysis of VEGF levels in supernatants from TGFβ1-stimulated RPE cell cultures (*n* = 7). **E**. Predicted binding sequences of miR-15a-5p and VEGF. **F**. Luciferase reporter assay in HEK293 cells cotransfected with pmirGLO–VEGF–3′UTR-WT or pmirGLO–VEGF–3′UTR–mutant (MUT) and agomir-15a-5p or agomir-NC (*n* = 4). Firefly luciferase activity reflects miRNA regulation. Renilla luciferase activity was used for normalization. **G–H**. Representative western blot images (**G**) and corresponding quantification (**H**) showing that agomir-15a-5p decreases VEGF protein expression in OIR retinas (*n* = 6). **I**. Quantitative PCR analysis showing that agomir-15a-5p reduces VEGF mRNA levels in the retina–choroid complex of CNV mice (*n* = 4). Data in **A, C, D, F, H,** and **I** are presented as mean ± SD and were compared using one-way ANOVA (**A**, **C, D, H, I**) or an unpaired two-tailed Student’s *t*-test (**F**). The original Western blot images used for quantification in this study have been deposited in figshare and can be accessed at DOI: 10.6084/m9.figshare.30820226.

Candidate genes regulated by miR-15a-5p were identified using the miRecords, miRTarBase, and TarBase databases, followed by pathway enrichment analysis using Kyoto Encyclopedia of Genes and Genomes (KEGG). Among the enriched pathways, several were associated with angiogenesis and TGFβ signaling, which was consistent with our experimental observations (**Figure S9**). Notably, miR-15a-5p was predicted to directly target VEGF, a key proangiogenic factor. We then validated this regulatory relationship in RPE cells using a TGFβ1-induced model that promotes VEGF secretion. Under TGFβ1 stimulation, transfection with miR-15a-5p markedly reduced VEGF expression (**Figure 6A–C**). ELISA of the culture supernatant confirmed that miR-15a-5p suppressed VEGF secretion (**Figure 6D**). Dual-luciferase reporter analysis showed that miR-15a-5p selectively suppressed the activity of the reporter carrying the wild-type VEGF 3′UTR, whereas disruption of the predicted binding site eliminated this response (**Figure 6E–F**). Consistently, agomir-15a-5p reduced VEGF expression in both the OIR and CNV models (**Figure 6G–I**). These results show that miR-15a-5p directly regulates VEGF gene expression *in vitro* and in pathological retinal models.

**Figure 7:**
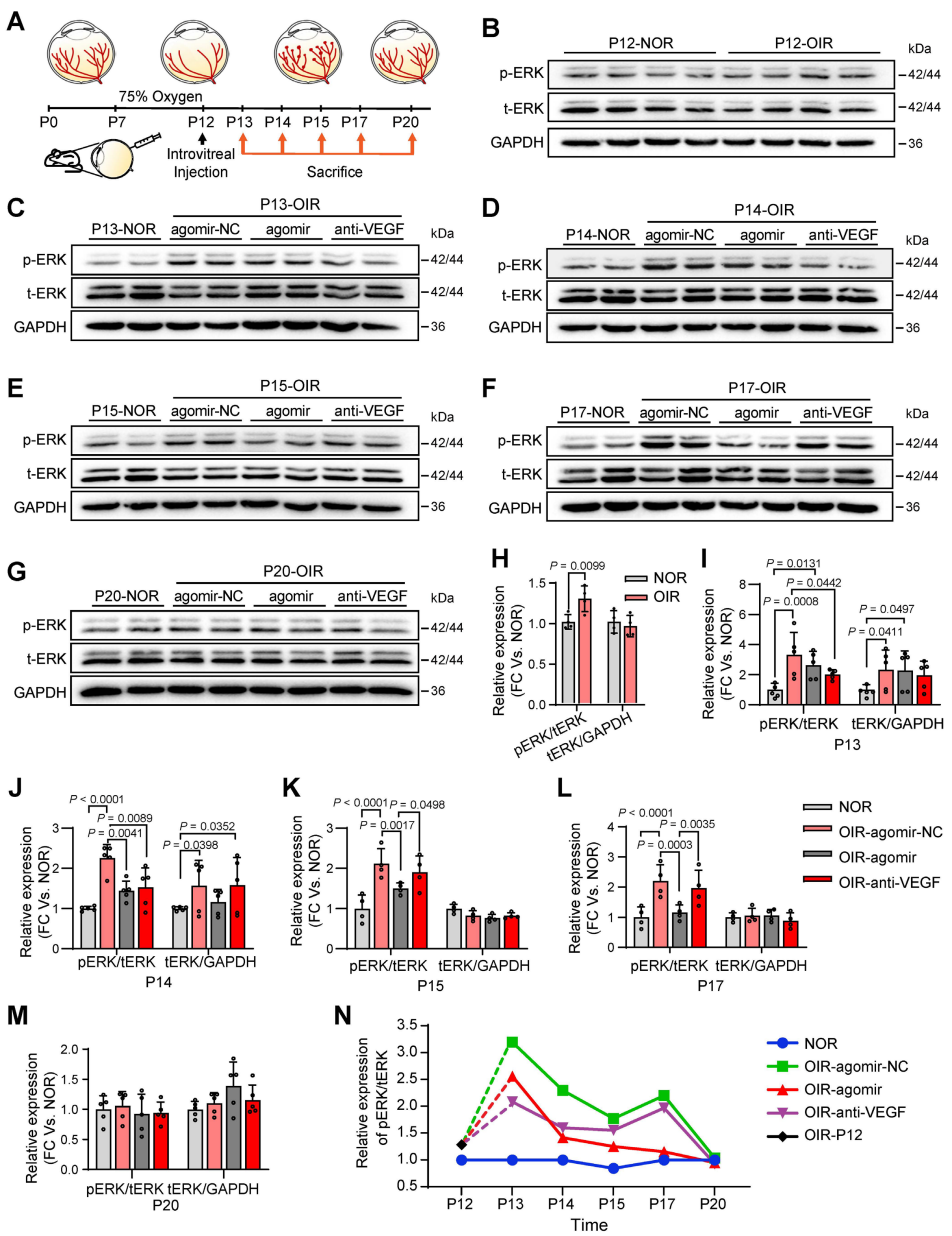
** miR-15a-5p induced more sustained ERK dephosphorylation in the retinas of OIR mice compared to anti-VEGF therapy. A**. Schematic diagram illustrating the experimental timeline. Retinas were harvested from mice at postnatal days P12, P13, P14, P15, P17, and P20. Intravitreal injections of agomir-NC, agomir-15a-5p, or anti-VEGF were administered at P12.** B**. Representative western blot images of phosphorylated ERK (p-ERK) and total ERK (t-ERK) in retinas from normal control (NOR) and OIR mice at P12 before any intravitreal injection.** C–G**. Representative western blot images of retinal p-ERK and t-ERK levels from NOR and OIR mice across different treatment groups (agomir-NC, agomir-15a-5p, anti-VEGF) at P13, P14, P15, P17, and P20, respectively. **H**. Quantitative analysis of the p-ERK/t-ERK ratio in NOR and OIR retinas at P12 (corresponding to B) (*n* = 4).** I–M**. Quantitative analysis of the p-ERK/t-ERK ratio across the different treatment groups at P13, P14, P15, P17, and P20, respectively (corresponding to **C–G**) (*n* = 4–5). **N**. Time-course line graph of the p-ERK/t-ERK ratio, normalized to the NOR group at P12, demonstrating the kinetic profiles of ERK phosphorylation suppression following treatments. Data in** H** are presented as mean ± SD and were analyzed using an unpaired two-tailed Student’s *t*-test. Data in** I, J, K, L, M** are presented as mean ± SD and were compared using one-way ANOVA. Data in **N** are presented as mean. Statistical analysis was performed using two-way repeated-measures ANOVA followed by Šidák’s multiple-comparisons test. A significant main effect of group was observed (F = 11.115, *P* < 0.0001), whereas the main effect of time was not significant (F = 0.080, *P* = 0.988). A significant group × time interaction was detected (F = 2.557, *P* = 0.0106). The original Western blot images used for quantification in this study have been deposited in figshare and can be accessed at DOI: 10.6084/m9.figshare.30820226.

Given that VEGF exerts proangiogenic effects largely through intracellular signaling cascades, we sought to determine whether the functional similarities between these two treatments extended to the modulation of a key downstream pathway. Therefore, we performed a detailed time-course analysis of ERK phosphorylation. As shown in **Figure 7A**, retinas were harvested from P12 to P20 after a single intravitreal injection at P12. Prior to any intervention at P12, retinas exposed to 12 hours of normoxia exhibited significantly elevated p-ERK levels compared to NOR controls (**Figure 7B, H**), confirming hyperactivation of this pathway in the model. Subsequent analysis revealed a critical divergence; while the anti-VEGF drug elicited a rapid reduction in ERK phosphorylation, agomir-15a-5p induced a more gradual but remarkably sustained suppression (**Figure 7C–G, I–N**). These findings suggest that, by directly suppressing VEGF mRNA, miR-15a-5p achieves more sustained ERK pathway inactivation and consequently exerts longer-lasting downstream effects than the transient neutralization of VEGF protein by an anti-VEGF drug.

### miR-15a-5p directly represses Smad2 to attenuate retinal fibrosis and endothelial-to-mesenchymal transition (EndoMT)

**Figure 8:**
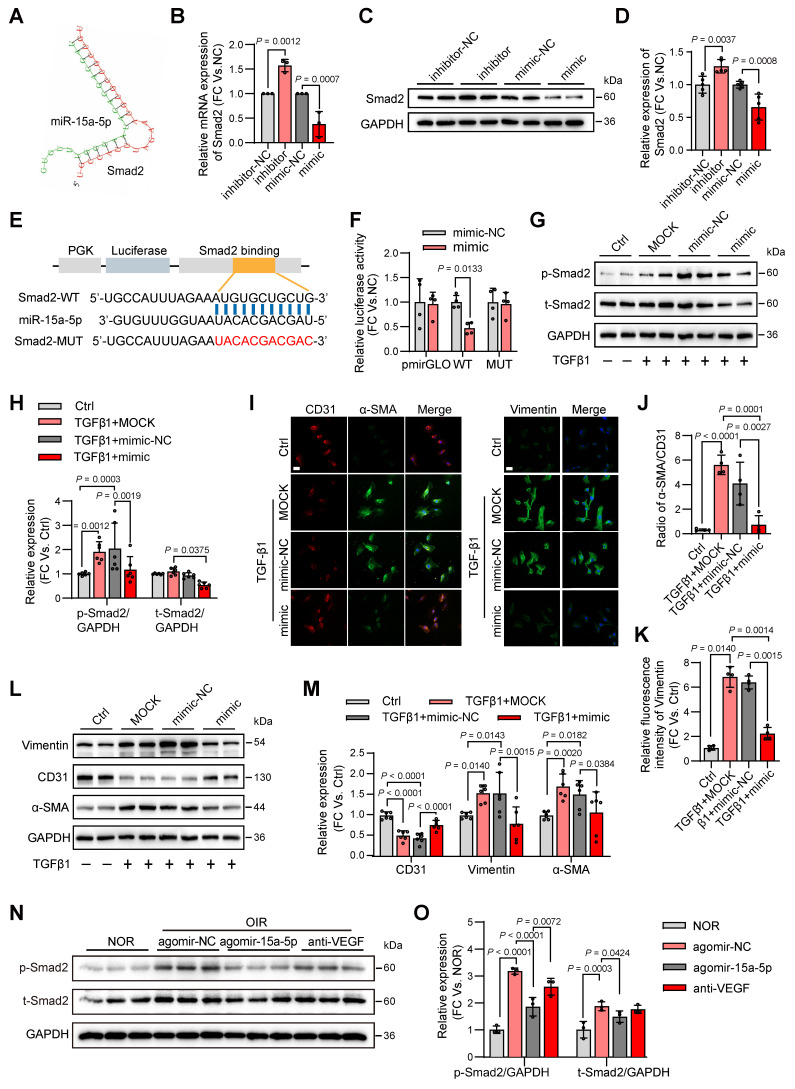
** miR-15a-5p directly regulated Smad2 expression and decreased EndoMT in HRMECs. A**. Schematic representation of the predicted base pairing between miR-15a-5p and the Smad2 3′UTR. **B**. Quantitative PCR analysis showing that miR-15a-5p suppresses Smad2 mRNA expression in HRMECs (*n* = 3). **C–D**. Representative western blot images (**C**) and corresponding quantification of Smad2 protein levels (**D**) in HRMECs (*n* = 5). **E**. The predicted binding sequences of miR-15a-5p and Smad2. **F**. Luciferase reporter assay in HEK293 cells transfected with pmirGLO–Smad2–3′UTR-WT or pmirGLO–Smad2–3′UTR–MUT and agomir-15a-5p or agomir-NC (*n* = 4). Firefly luciferase activity reflects miRNA regulation, and Renilla luciferase activity was used for normalization.** G**. Representative western blot images of p-Smad2 and t-Smad2 in HRMECs under TGFβ1-induced EndoMT conditions. **H**. Quantitative analysis of the p-Smad2 and t-Smad2 protein levels (*n* = 6).** I**. Immunofluorescence staining for CD31 (endothelial marker, red) and α-SMA (fibrotic marker, green) in HRMECs (left panel). Immunofluorescence staining for Vimentin (fibrotic marker, green) in HRMECs (right panel). Blue indicates DAPI staining. Scale bar: 20 μm. **J**. Quantification of the α-SMA/CD31 fluorescence intensity ratio in different groups (*n* = 4). **K**. Quantification of the relative fluorescence intensity of vimentin in different groups (*n* = 4). **L**. Representative western blot images of vimentin, CD31, and α-SMA in HRMECs under TGFβ1-induced EndoMT conditions. **M**. Quantitative analysis of protein expression levels for vimentin, CD31, and α-SMA (*n* = 6). **N.** Representative western blot images showing p-Smad2 and t-Smad2 expression in retinas from OIR mice at P17. **O.** Quantitative analysis of the p-Smad2 and t-Smad2 protein levels (*n* = 3). Data in **B, D, F, H, J, K, M,** and **O** are presented as mean ± SD and analyzed using one-way ANOVA (**B, D, H, J, K, M,** and **O**) or an unpaired two-tailed Student’s *t*-test (**F**). The original Western blot images used for quantification in this study have been deposited in figshare and can be accessed at DOI: 10.6084/m9.figshare.30820226.

Besides VEGF, multiple in silico predictions using miRecords, miRTarBase, and TarBase together with KEGG pathway enrichment analysis results (**Figure S9**) suggested that miR-15a-5p may regulate components of the TGFβ signaling pathway. Given the central role of Smad2 as a downstream effector, we examined whether miR-15a-5p directly targets Smad2. Predicted base pairing between miR-15a-5p and the Smad2 3′UTR was analyzed (**Figure 8A**). Smad2 expression was markedly suppressed after miR-15a-5p overexpression (**Figure 8B–D**). Reporter analysis supported a direct interaction with the Smad2 3′UTR, as only the intact construct exhibited reduced reporter activity, whereas disruption of the predicted binding site eliminated this response (**Figure 8E–F**). miR-15a-5p alleviated the TGFβ1-induced mesenchymal phenotype by decreasing α-SMA and vimentin, while restoring CD31 expression (**Figure 8G–M**). These results indicated that miR-15a-5p directly repressed Smad2 and inhibited EndoMT in endothelial cells. Retinal fibrosis was initially characterized by western blot analysis of fibrotic markers in OIR mice. Specifically, at P17, OIR retinas demonstrated activation of Smad2 signaling along with the overexpression of key fibrotic proteins, including fibronectin, TGFβRII, and α-SMA (**Figure S10**). In OIR retinas, miR-15a-5p treatment decreased Smad2 phosphorylation (**Figure 8N–O**). In parallel, immunofluorescence staining revealed reduced α-SMA and fibronectin accumulation following miR-15a-5p treatment, while anti-VEGF therapy showed minimal effect (**Figure S11A–C**). Western blot analysis confirmed decreased fibrotic protein levels, including fibronectin, α-SMA, and TGFβRII (**Figure S11D–F**).

In the CNV model, α-SMA staining was used to visualize fibrotic changes within the lesion area, revealing evident fibrosis associated with choroidal neovascularization (**Figure S12A**). Only agomir-15a-5p significantly reduced fibrotic signaling around neovascular lesions, whereas anti-VEGF treatment showed no such effect (**Figure S12B**). Consistently, miR-15a-5p KO mice exhibited exacerbated neovascular lesions and fibrosis compared to WT mice, both of which were effectively reversed by agomir-15a-5p administration (**Figure S12C–D**). Overall, these findings suggest that miR-15a-5p mediates the reduction of retinal fibrosis, at least in part, through the direct repression of Smad2 and downstream fibrotic signaling.

### miR-15a-5p exhibits superior systemic and ocular safety compared with the anti-VEGF drug

To evaluate the systemic and ocular safety of miR-15a-5p administration, we first examined normal mice that had received intravitreal agomir-15a-5p or anti-VEGF drug injections. Mice in the anti-VEGF group showed reduced body weight (**Figure S13A**) and elevated triglyceride levels (**Figure S13B**), whereas other serum biochemical parameters remained within the normal ranges (**Figure S13C–E**). Following the intravitreal injection of Cy3-labeled agomir-15a-5p, no Cy3 fluorescence was observed in the liver or kidney at any of the examined time points (**Figure S13F**), and histological analysis revealed no structural abnormalities in these organs (**Figure S13G–H**). Coagulation and urinary protein levels in mice remained within the normal range (**Figure S13I–J**). These results suggested minimal systemic dissemination of agomir-15a-5p. Retinal morphology analysis showed that agomir-15a-5p caused no structural changes, whereas anti-VEGF treatment induced mild retinal thinning (**Figure S14A–C**). No intraocular drug injection was found to elevate retinal GFAP expression (**Figure S14D–E**). Fundus fluorescein angiography and electroretinography further confirmed that intravitreal administration of agomir-15a-5p did not alter retinal vascular morphology or retinal function in normal mice (**Figure S14F–G**). Furthermore, retinal inflammation levels were not increased after either single or repeated intravitreal injections of agomir-15a-5p (**Figure S14H–J**). Collectively, these results indicate a favorable systemic and ocular safety profile of miR-15a-5p under physiological conditions.

Next, we compared the systemic safety of the two agents in OIR mice. Throughout the observation period, OIR mice exhibited lower body weights than normal controls; however, the treatment groups exhibited comparable responses (**Figure S15A**). Serum from anti-VEGF-treated OIR mice appeared milky (**Figure S15B**), with significantly elevated triglyceride and blood urea nitrogen levels persisting until P25 (**Figure S15C, D**). The OIR group exhibited significantly decreased creatinine levels, which may be associated with the lower body weight in these mice (**Figure S15E**). In addition, the serum total cholesterol levels of the OIR group were decreased relative to those in NOR mice at P17 (**Figure S15F**). Histological analyses of liver and kidney tissues revealed no obvious histopathological abnormalities (**Figure S15G, H**). The more pronounced systemic alterations observed in the OIR anti-VEGF group may have resulted from blood–retinal barrier disruption, facilitating drug leakage into circulation. Moreover, the intravitreal administration of miR-15a-5p did not increase retinal apoptosis (**Figure S16**). Overall, these data suggest that miR-15a-5p exhibits better systemic and ocular safety than the anti-VEGF drug, even under pathological conditions.

## Discussion

In this study, we demonstrated that miR-15a-5p is a key regulator of retinal neovascularization, surpassing the efficacy of conventional anti-VEGF therapy by simultaneously inhibiting fibrosis and glial activation and promoting retinal neuroprotection while maintaining an improved safety profile. miR-15a-5p targets VEGF to achieve sustained suppression of downstream signaling and Smad2 to reduce retinal fibrosis and EndoMT. These results imply that miR-15a-5p could be a promising therapeutic agent for ocular neovascularization and fibrosis.

miRNAs are small noncoding RNA molecules. Their encapsulation in sEVs helps stabilize them in circulation, allowing them to participate in a variety of cellular processes 14-16. Previous studies have revealed diverse roles of sEV-derived miRNAs in the pathogenesis of neovascular diseases. Xiong *et al*. presented evidence that miR-20b-5p in plasma-derived sEVs from diabetic subjects can delay angiogenesis and wound healing 17. miR-431-5p is upregulated in sEVs from PDR patients and contributes to abnormal endothelial cell proliferation 9. However, these studies did not examine whether plasma-derived sEVs and intraocular fluids exhibit similar changes in miRNA profiles. Our analysis revealed a marked increase in miR-15a-5p expression in both plasma-derived sEVs and vitreous humor. Although the precise cellular origin of miR-15a-5p remains unclear, previous reports have suggested that circulating extracellular vesicles largely originate from platelets, monocytes, and macrophages 18. Notably, increased miR-15a-5p expression has been reported in monocyte-derived microvesicles following GM-CSF stimulation 19. Under diabetic conditions, disruption of the blood–retinal barrier may facilitate the infiltration of circulating sEVs to the vitreous humor. In line with our findings, a recent study also reported elevated miR-15a-5p levels in vitreous-derived exosomes from PDR patients 20. Overall, the enhanced expression of miR-15a-5p in intraocular fluids and circulating sEVs emphasizes its possible involvement in the pathogenic development of DR.

Interestingly, miR-15a-5p was moderately upregulated in the intraocular fluids of patients during the natural course of retinal neovascularization. Similar increases have been reported for other endogenous anti-angiogenic mediators, including PEDF, TSP-2, and miR-143-3p 21-25. However, these endogenous protective responses are evidently insufficient to prevent the progression of retinal neovascularization. This observation may reflect the persistent pathological burden imposed by diabetes, which exceeds the protective capacity of endogenous anti-angiogenic mediators 26. Moreover, the sustained disruption of angiogenic homeostasis in the diabetic retina may limit the effectiveness of these endogenous protective responses 27. Consistent with this interpretation, the therapeutic benefits observed following early miR-15a-5p supplementation suggest that exogenous delivery of miR-15a-5p may help overcome the limited protective potential of the endogenous response.

Given the role of miR-15a-5p in angiogenic regulation, we proposed that it might also be essential for maintaining vascular homeostasis. A previous study reported that endothelial-specific deletion of miR-15a-5p resulted in exacerbated retinal leukostasis accompanied by elevated levels of inflammatory cytokines 11. Consistently, miR-15a-5p knockout mice in our study exhibited retinal abnormalities, including inner plexiform layer disruption and reduced electrophysiological amplitudes. The vascular abnormalities observed following miR-15a-5p deficiency during development suggest that miR-15a-5p is important for retinal vascular morphogenesis. In contrast, therapeutic-dose supplementation of miR-15a-5p did not induce obvious retinal toxicity in normal adult mice, indicating a tolerable therapeutic window in the mature retina. Together, these findings identify miR-15a-5p as an important regulator of retinal vascular homeostasis and angiogenesis.

Beyond retinal avascularity and neovascular tufts, the OIR and CNV models also exhibited significant fibrotic remodeling. Ma *et al*. reported significant upregulation of the profibrotic marker α-SMA and fibronectin in OIR retinas, associated with elevated TGF-β/Smad2/3 signaling 28. These findings are consistent with the fibrotic changes we observed in the retinas of OIR mice. Importantly, we found that although anti-VEGF therapy was effective in suppressing neovascularization, it had a limited impact on fibrotic changes in OIR and CNV models. A body of clinical evidence has indicated that anti-VEGF agents are not optimal for treating stage 4–5 ROP, as they can exacerbate fibrovascular proliferation and precipitate the so-called “ROP crunch” 29, 30. Indeed, studies have identified increased levels of fibrosis-related proteins in the vitreous of PDR patients following anti-VEGF treatment 31, 32. Studies have shown that hyperglycemia can induce EndoMT by activating TGF-β, Wnt/β-catenin, and Notch signaling pathways 33-35. For example, miR-9 can inhibit early neovascularization by altering the TGF-β/Smad pathway 36. Similarly, Zhao *et al*. found that miR-15a-5p reduces fibrosis and associated inflammation in human peritoneal mesothelial cells and the peritoneum 37, 38. In the current study, we further assessed the antifibrotic and anti-inflammatory effects of miR-15a-5p in OIR and CNV models and provided direct evidence that it targets Smad2 to inhibit retinal fibrosis and EndoMT, providing mechanistic insight into its protective role in pathological neovascularization and fibrosis.

Antibody-based therapeutics, such as anti-VEGF therapy, act rapidly by neutralizing ligands or blocking receptors, which, in our study, caused strong early suppression of downstream signaling. However, their effects are often transient and limited by acquired resistance or feedback activation, resulting in partial recovery of signaling 39. In this study, although both treatments ultimately achieved comparable suppression of neovascularization in the OIR model, miR-15a-5p may yield more favorable outcomes in disease settings characterized by sustained VEGF elevation. Antibody-based therapies often exhibit a relatively short duration of action and require repeated administration, which may increase the risk of intraocular infection. In contrast, miRNA-based therapeutics generally have a slower onset of action but a more sustained effect, which may help reduce dosing frequency. Recent studies have shown that polymeric sustained-release microspheres can enable long-term delivery of anti-VEGF agents; however, owing to the large molecular size of antibodies, drug-loading efficiency in such delivery systems remains limited 40. In contrast, the smaller molecular size of miRNAs may facilitate their incorporation into emerging nanomaterial-based delivery platforms, providing additional opportunities for sustained intraocular delivery.

RNA-based therapeutics have already been approved for treating several diseases worldwide, and multiple clinical trials verifying their applicability and safety are ongoing 41, 42. Recently, the world’s first small interfering RNA therapy targeting low-density lipoprotein cholesterol has been approved, providing compelling evidence for the clinical viability of RNA-based therapeutics 43. In ophthalmology, anti-miR-132 has demonstrated potent anti-angiogenic effects in preclinical models, highlighting its potential as a therapeutic strategy for neovascular retinal diseases 44, 45. In contrast to miRNA candidates that primarily target pathological angiogenesis, our findings suggest that miR-15a-5p may have a dual-target therapeutic effect by inhibiting both neovascularization and retinal fibrosis. This broader activity may offer additional neuroprotective benefits and address multiple pathological processes contributing to vision loss. Despite the growing translational potential of miRNA-based therapeutics, several challenges remain before clinical implementation. Efficient and targeted *in vivo* delivery remains a major obstacle, as naked miRNAs are susceptible to rapid degradation and exhibit limited tissue penetration. In addition, because a single miRNA can regulate multiple downstream targets, unintended off-target effects may occur and potentially affect normal cellular functions. Recent advances in lipid nanoparticle-based targeted delivery systems and biomaterial-based carriers, such as extracellular vesicles, may improve the stability, delivery efficiency, and safety of miRNA therapeutics 46-48. Future studies should include comprehensive assessments of long-term biosafety, possible non-specific effects, and the impact of miR-15a-5p on physiological angiogenesis in relevant models, which will be important for facilitating the clinical translation of miRNA-based therapeutics.

Despite these promising findings, this study has several limitations. First, the expression of miR-15a-5p in retinal neurons, its direct neuroprotective effects, and the underlying neuronal target genes require further investigation. Second, lineage-tracing approaches were not employed to definitively validate the endothelial origin of mesenchymal-like cells during EndoMT. Addressing these limitations in future studies will provide further insight into the mechanisms underlying miR-15a-5p-mediated retinal protection.

## Conclusions

In conclusion, our findings indicate that miR-15a-5p inhibits pathological neovascularization to a similar extent as anti-VEGF therapy while also providing vascular normalization, retinal neuroprotection, and a potentially improved safety profile. These properties make miR-15a-5p a promising therapeutic candidate for retinal vascular disorders. Future studies focusing on optimized delivery systems and their long-term efficacy in large animal models will advance their clinical translation.

## Supplementary Material

Supplementary figures and tables.

## Data Availability

All original uncropped western blot images are available on figshare (10.6084/m9.figshare.30820226). Detailed catalog numbers for all reagents and consumables used in this study are listed in Supplementary Table 4. The data supporting the findings of this study are available from the corresponding author upon reasonable request. For data access, please contact lixiaorong@tmu.edu.cn.

## Abbreviations

wAMD: wet age-related macular degeneration
PDR: proliferative diabetic retinopathy
VEGF: vascular endothelial growth factor
miRNAs: microRNAs
EVs: extracellular vesicles
sEVs: small extracellular vesicles
m/lEVs: medium/large extracellular vesicles
DR: diabetic retinopathy
AH: aqueous humor
ROC: receiver operating characteristic
HRMECs: human retinal microvascular endothelial cells
KO: knockout
WT: wild-type
OCT: optical coherence tomography
ERG: electroretinography
OIR: oxygen-induced retinopathy
CNV: choroidal neovascularization
H&E: hematoxylin and eosin
RGC: retinal ganglion cell
IPL: inner plexiform layer
INL: inner nuclear layer
OPL: outer plexiform layer
ONL: outer nuclear layer
RPE: retinal pigment epithelium
ICAM-1: intercellular adhesion molecule-1
KEGG: Kyoto Encyclopedia of Genes and Genomes
EndoMT: endothelial-to-mesenchymal transition
