# Supplementary material for: miR-15a-5p outperforms anti-VEGF drug in ocular neovascularization by providing dual anti-angiogenic and neuroprotective effects
Source: Theranostics. 2026 Jul 29;16(14):8427–46. doi: 10.7150/thno.134644 (PMC13440569; doi:10.7150/thno.134644)
Supplement: Supplementary file 1 [file thnov16p8427s1.pdf]

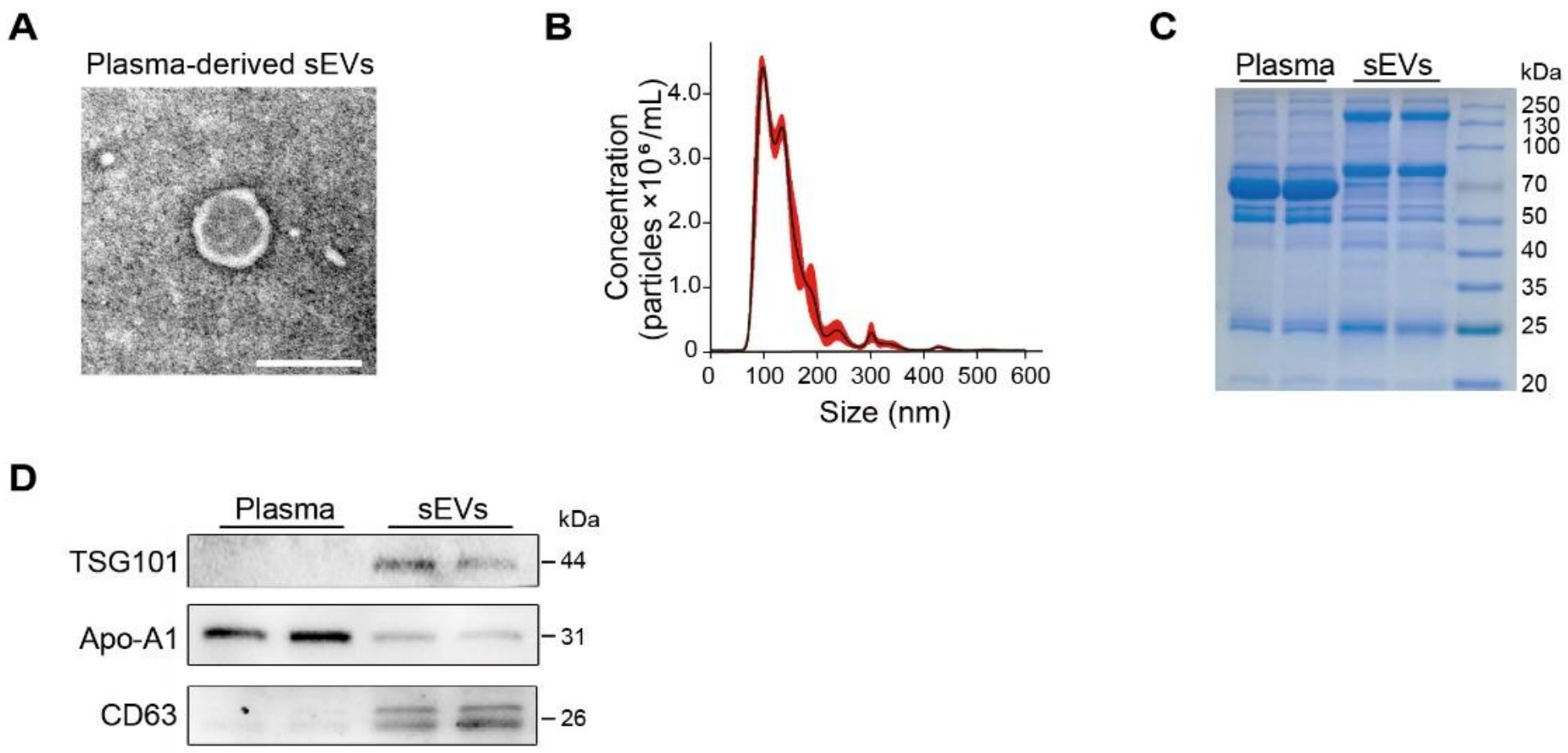

Supplementary Figure 1. Characterization of plasma-derived sEVs. A. Representative transmission electron microscope image of sEVs. Scale bar: 200 nm. **B**. Nanoparticle tracking analysis, showing particle size distribution of plasma-derived sEVs. **C**. Protein bands of sEVs and plasma, visualized with Coomassie Brilliant Blue. **D**. Western blotting analysis of plasma and sEVs. Equal protein loading was used as a blotting control. TSG101 and CD63 are exosome markers. Apo-A1 was used as a purity control. The original Western blot images used for quantification in this study have been deposited in figshare and can be accessed at DOI: 10.6084/m9.figshare.30820226.

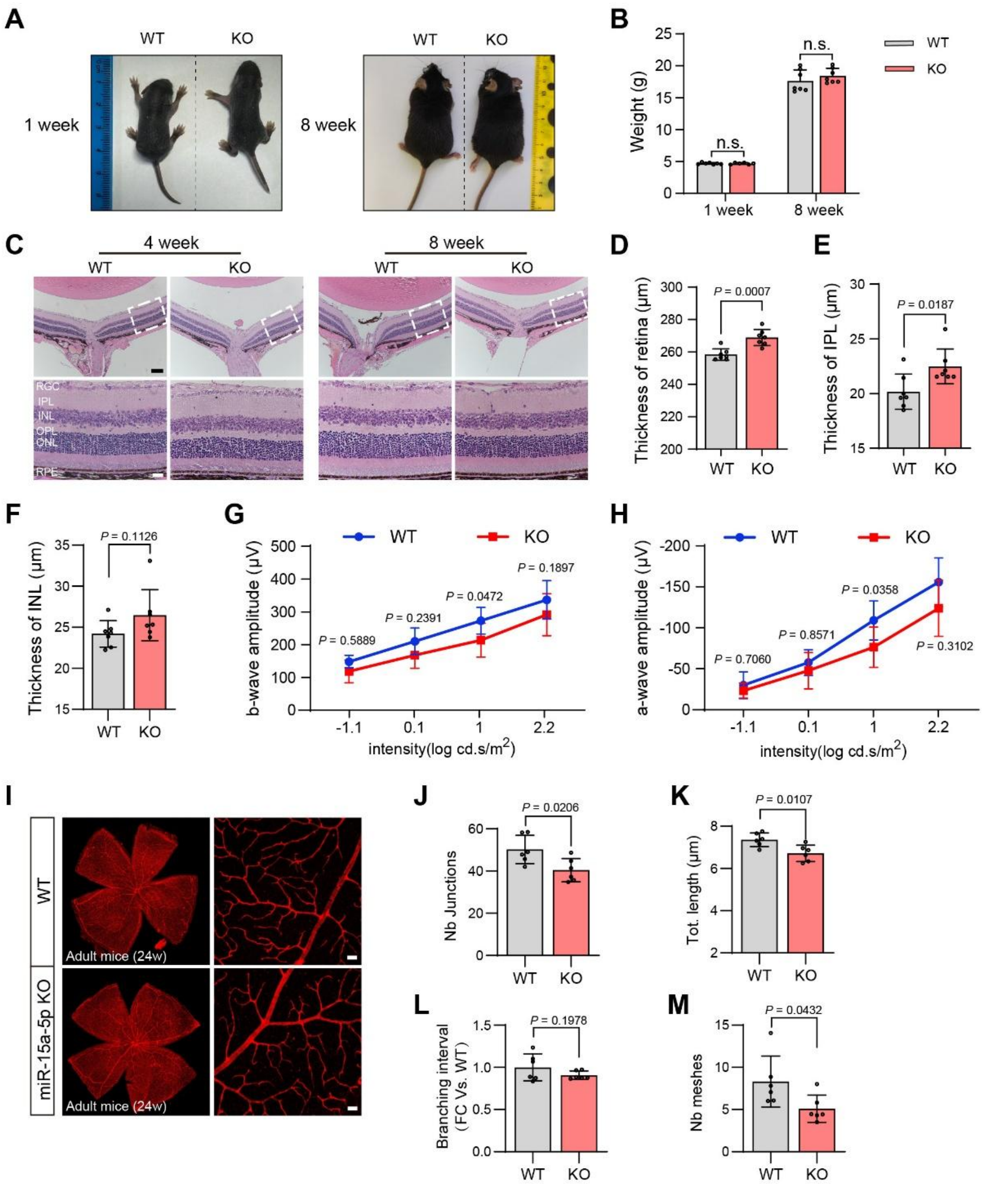

Supplementary Figure 2. Effects of miR-15a-5p deficiency on physical growth and retinal structure and function in mice. A. Comparisons of body size from WT controls and miR-15a-5p KO mice at 1 week and 8 weeks. **B**. Body weight of pups and adult mice from WT and KO mice (*n* = 7). **C**. Representative images of retinal sagittal sections from WT and KO mice at 4 weeks (left) and 8 weeks (right) of age. Scale bar: 100 μm (upper panel), 20 μm (bottom panel). **D**. Quantitative analysis of retinal thickness in WT and KO mice at 8 weeks of age (*n* = 7). **E**–

F. Quantitative analysis of IPL (**E**) and INL (**F**) thickness in WT and KO mice at 8 weeks of age (*n* = 7). **G–H**. Quantification of ERG b-wave (**G**) and a-wave (**H**) amplitudes in WT and KO mice at 8 weeks of age (*n* = 8). **I.** Representative IB4-stained retinal whole mounts from adult WT and KO mice showing vascular morphology. Scale bar: 50 μm. **J–M**. Quantification of retinal vascular parameters, including junction number (**J**), total vessel length (**K**), branch interval (**L**), and total mesh number (**M**). Abbreviations: RGC, retinal ganglion cell layer; IPL, inner plexiform layer; INL, inner nuclear layer; OPL, outer plexiform layer; ONL, outer nuclear layer; RPE, retinal pigment epithelium. Data in **B, D, E, F, G, H, J, K, L,** and **M** are presented as mean ± SD and analyzed using an unpaired two-tailed Student's *t*-test.

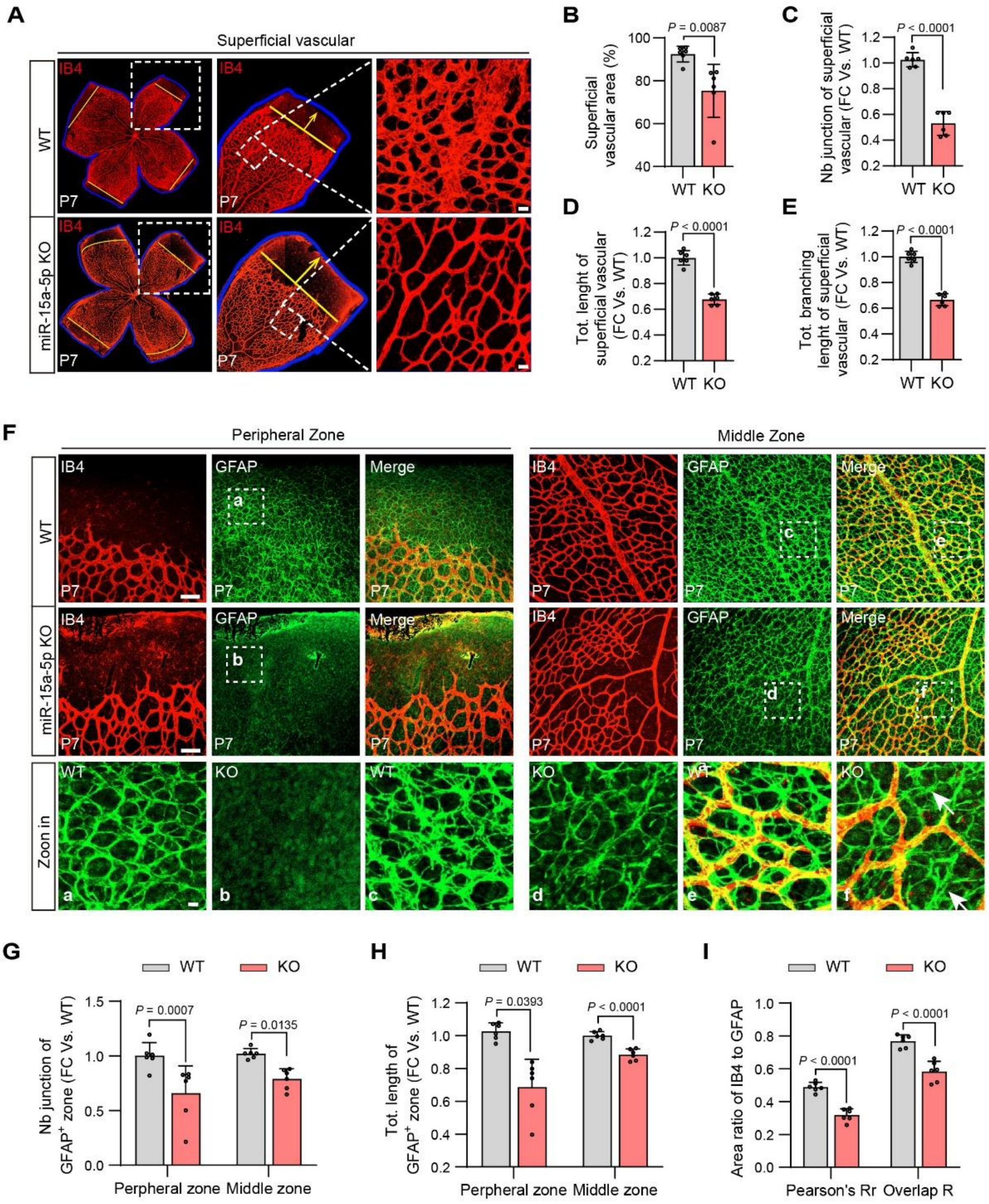

Supplementary Figure 3. miR-15a-5p deficiency disrupts astrocyte network organization and impairs superficial retinal vascular development. A. Representative images of retinal vasculature labeled with fluorescence-conjugated IB4 in KO and WT mice at P7. The blue outline indicates the retinal boundary, and the yellow line marks the front of the developing superficial vascular plexus. Scale bar: 20 μm. **B**. Quantification of superficial retinal vascular coverage in KO and WT mice at P7 (*n* = 6). **C**. Quantitative analysis of the number of vascular

junctions in the superficial retinal layer (*n* = 6). **D**. Quantification of the total length of superficial retinal vessels (*n* = 6). **E**. Quantification of the total branch length of superficial retinal vessels (*n* = 6). **F**. Representative images of the peripheral (left) and middle (right) retina stained with IB4 (red, marking blood vessels) and GFAP (green, marking astrocytes). Panels a-f show higher-magnification views of the boxed regions. White arrows indicate areas devoid of vascular perfusion despite the presence of astrocytes. Scale bar: 200 μm (upper and middle panels). Scale bar: 20 μm (bottom panel). **G**. Relative quantitative analysis of the number of branch points in the GFAP-positive area in KO and WT mice (*n* = 6). **H**. Relative quantitative analysis of the total length of the GFAP-positive area in KO and WT mice (*n* = 6). **I**. Quantification of the colocalization between IB4 and GFAP signals in KO and WT mice (*n* = 6). Data in **B, C, D, E, G, H,** and **I** are presented as mean ± SD and analyzed using an unpaired two-tailed Student's *t*-test.

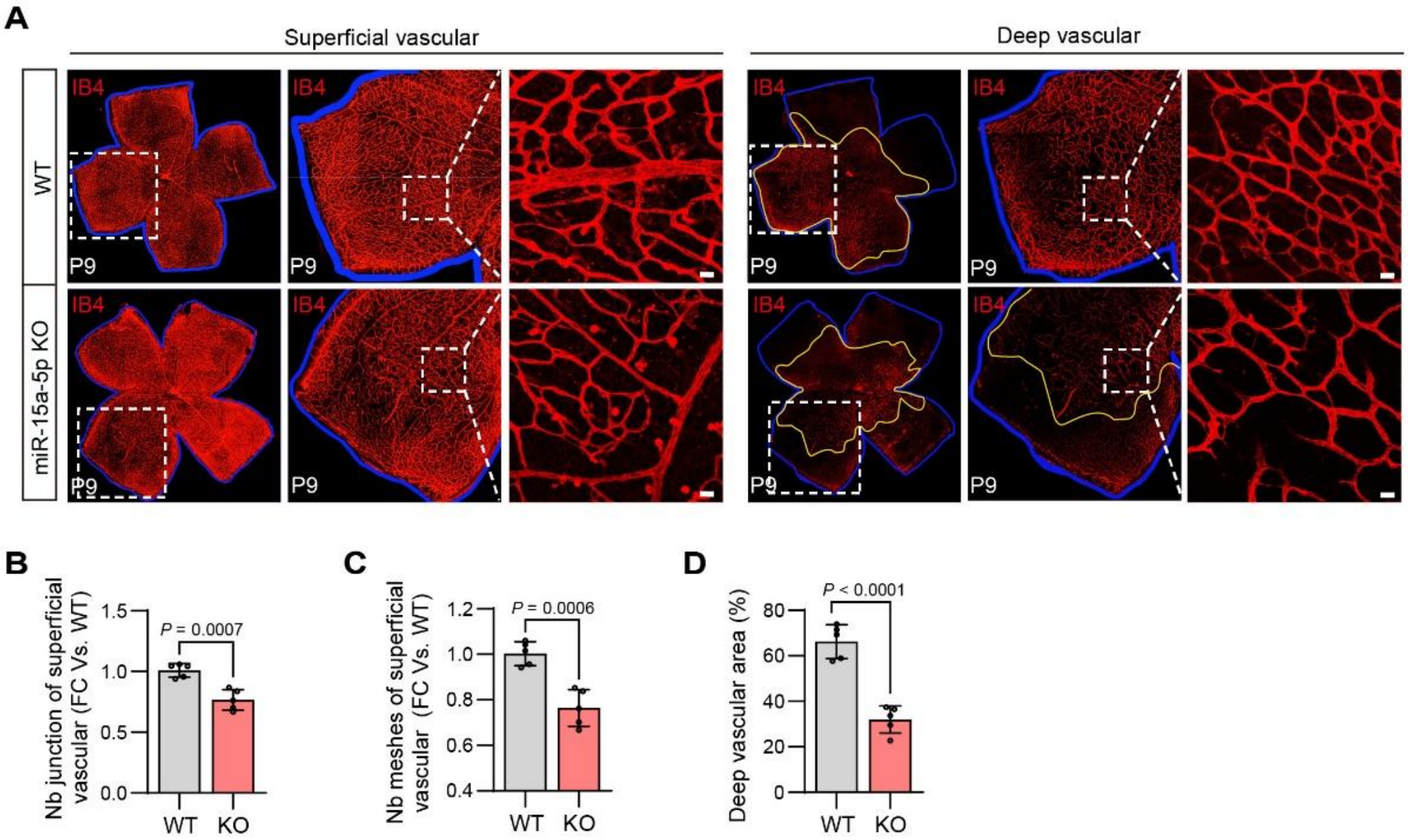

Supplementary Figure 4. miR-15a-5p deficiency delays deep retinal vascular development.

A. Representative images of retinal vasculature labeled with fluorescence-conjugated IB4 in KO and WT mice at P9. The blue outline indicates the retinal boundary. Scale bar: 20 μm. **B.** Quantitative analysis of the number of vascular junctions in the superficial retinal layer in KO and WT mice at P9 (*n* = 5). **C.** Quantitative analysis of the number of meshes in the superficial retinal layer in KO and WT mice at P9 (*n* = 5). **D.** Quantification of deep retinal vascular coverage in KO and WT mice at P9 (*n* = 5). Data in **B, C,** and **D** are presented as mean ± SD and analyzed using an unpaired two-tailed Student's *t*-test.

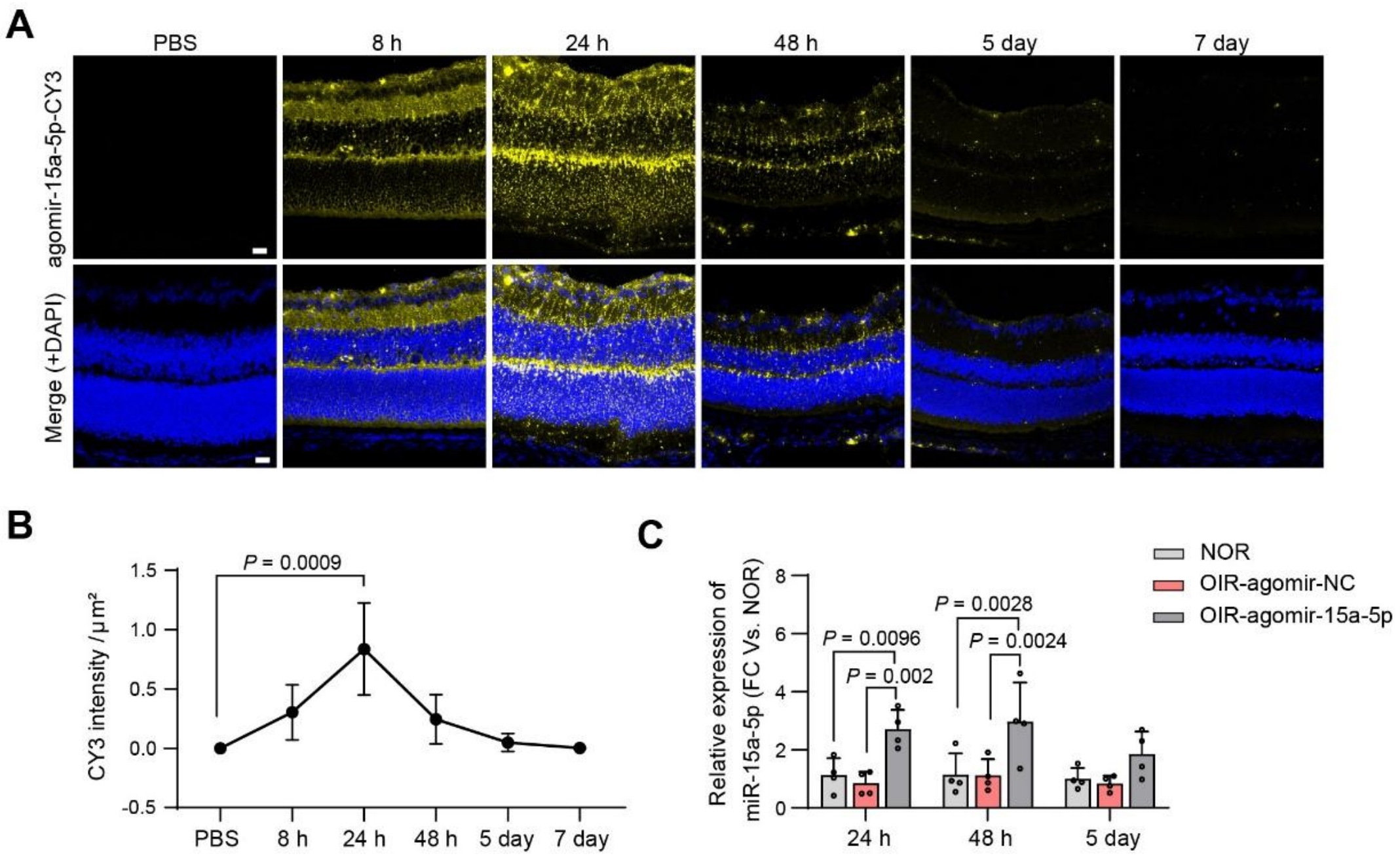

Supplementary Figure 5. Distribution and persistence of agomir-15a-5p in the retina after administration. A. Representative retinal images showing the localization of CY3-labeled agomir-15a-5p at indicated time points after intravitreal injection. Scale bar: 20 μm. **B**. Quantification of retinal CY3 fluorescence intensity after intravitreal injection at different time points (n = 3). **C**. Quantitative PCR analysis showing miR-15a-5p expression levels in retinas after intravitreal injection (n = 4). Data in **B** and **C** are presented as mean ± SD and are compared by one-way ANOVA.

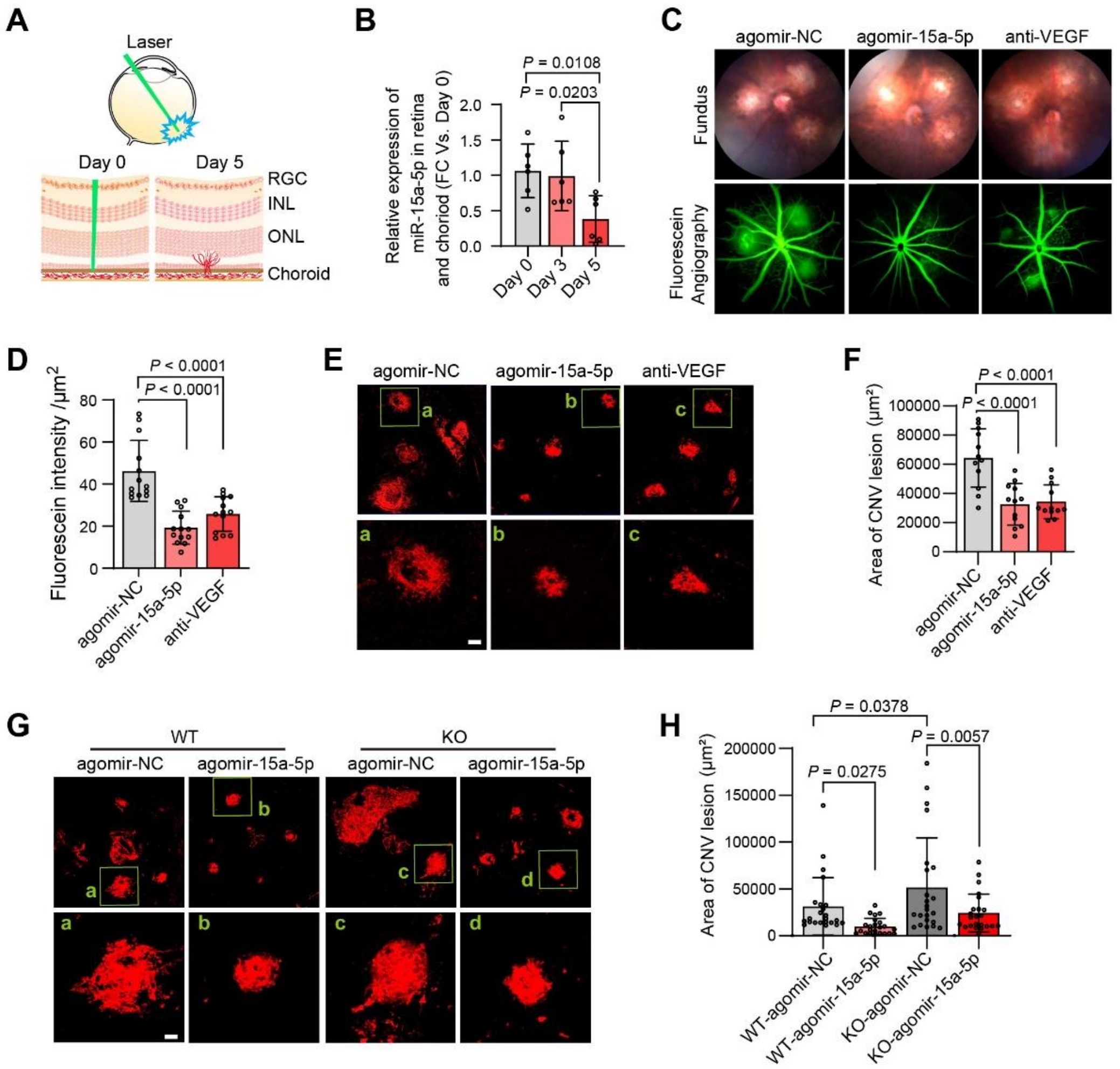

Supplementary Figure 6. miR-15a-5p administration suppresses choroidal neovascularization in the laser-induced CNV model. A. Schematic of the CNV mouse model. **B**. Quantitative RT-PCR analysis of miR-15a-5p levels in the retina and choroid of CNV mice (n = 6). **C**. Representative fundus photography and fluorescein angiography images showing CNV lesions in the retina. **D**. Quantification of fluorescein leakage area in the retina (n = 13 lesions from 4 mice for each group). **E**. Representative photos of IB4-labeled neovascular lesions in mice that underwent laser-induced CNV 5 days after laser exposure and treatment with agomir-15a-5p. Scale bar: 20 μm. **F**. Quantification of IB4-labeled neovascular lesions in the retina (n = 12 lesions from 4 mice for each group). **G**. Representative images of IB4-labeled neovascular lesions in KO and WT controls at 5 days after laser-induced CNV. Scale bar: 20 μm.

H. Quantification of IB4-labeled neovascular lesions in KO and WT controls (n = 23 lesions from 7 mice per group). Only one eye per mouse was used for the experiment. Data in **B, D, F,** and **H** are presented as mean ± SD and analyzed by one-way ANOVA.

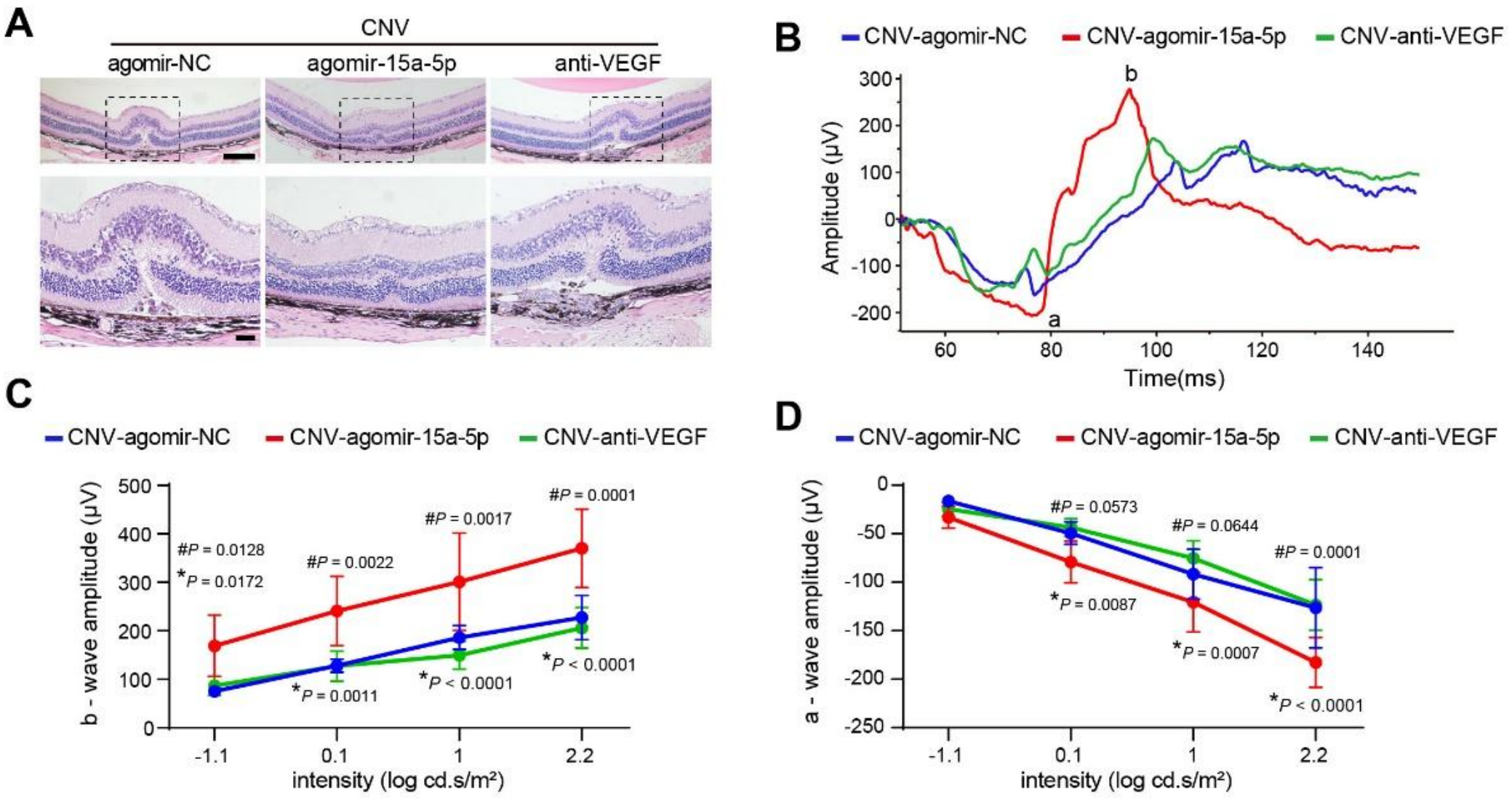

Supplementary Figure 7. Intravitreal injection of miR-15a-5p improves retinal structure and visual function in CNV mice and is superior to anti-VEGF treatment. A. Representative H&E-stained retinal sections from CNV mice. Scale bar: 100 µm (upper panel), 20 µm (bottom panel). **B**. Representative ERG waveforms of CNV mice in response to a 1.0 log cd·s/m² light flash. **C**. Quantitative analysis of b-wave amplitudes in CNV mice under different light intensities (n = 5-7). **D**. Quantitative analysis of a-wave amplitudes in CNV mice under different light intensities (n = 5-7). Data in **B, C** and **D** are presented as mean ± SD and analyzed using two-way ANOVA. # denotes a significant difference between agomir-15a-5p and agomir-NC; * denotes a significant difference between agomir-15a-5p and anti-VEGF.

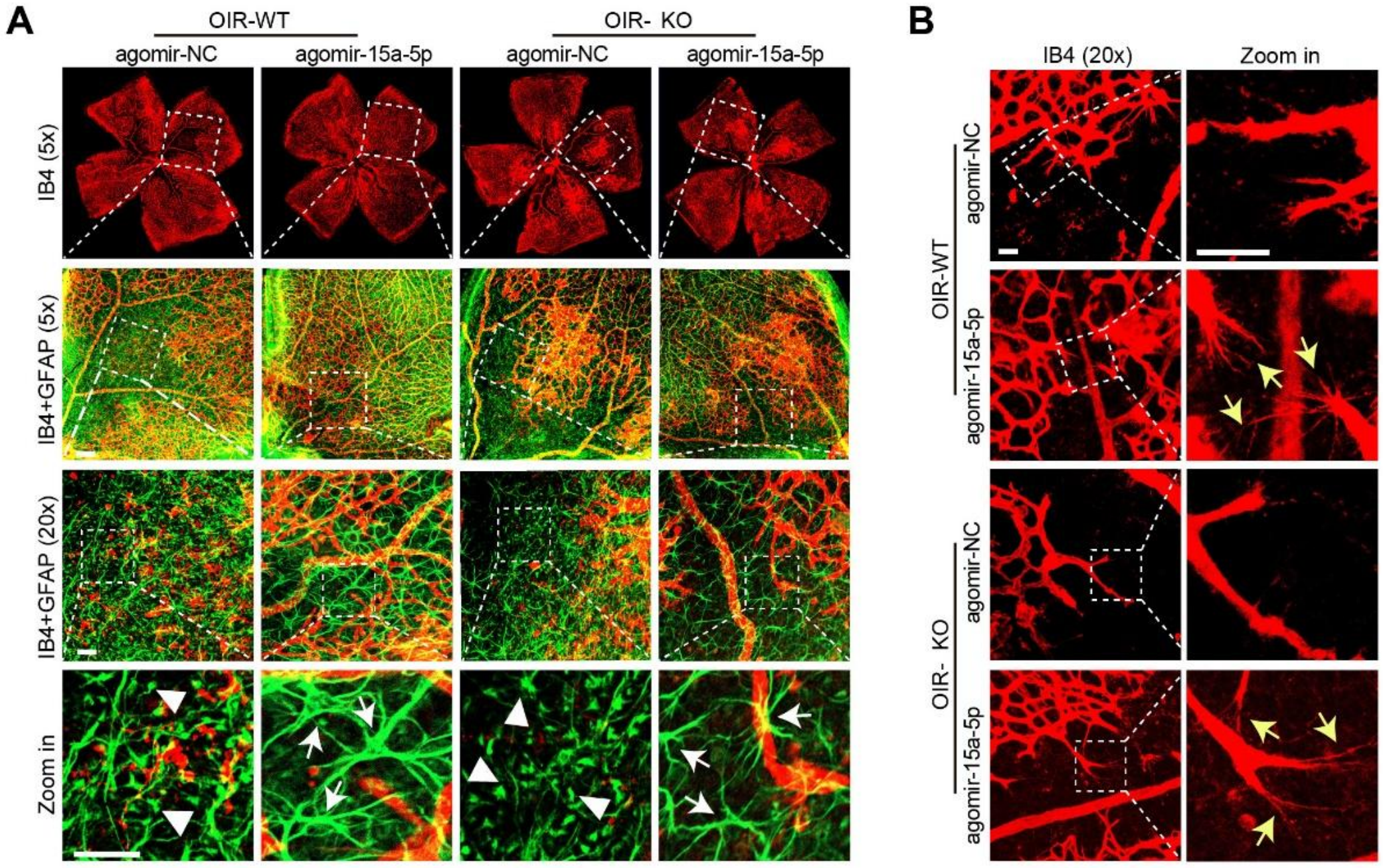

Supplementary Figure 8. miR-15a-5p deficiency disrupts astrocyte integrity and vascular remodeling, rescued by agomir-15a-5p. **A**. Representative images of non-perfused area margins showing astrocyte survival (white arrow) and Müller glia activation (white triangle) in WT controls and KO mice treated with agomir-NC or agomir-15a-5p. Scale bar: 80 μm (upper panel), 20 μm (middle and bottom panels). **B**. Representative images of retinal endothelial tip cells and filopodia (yellow arrow) in WT controls and KO mice treated with agomir-NC or agomir-15a-5p. Scale bar: 20 μm.

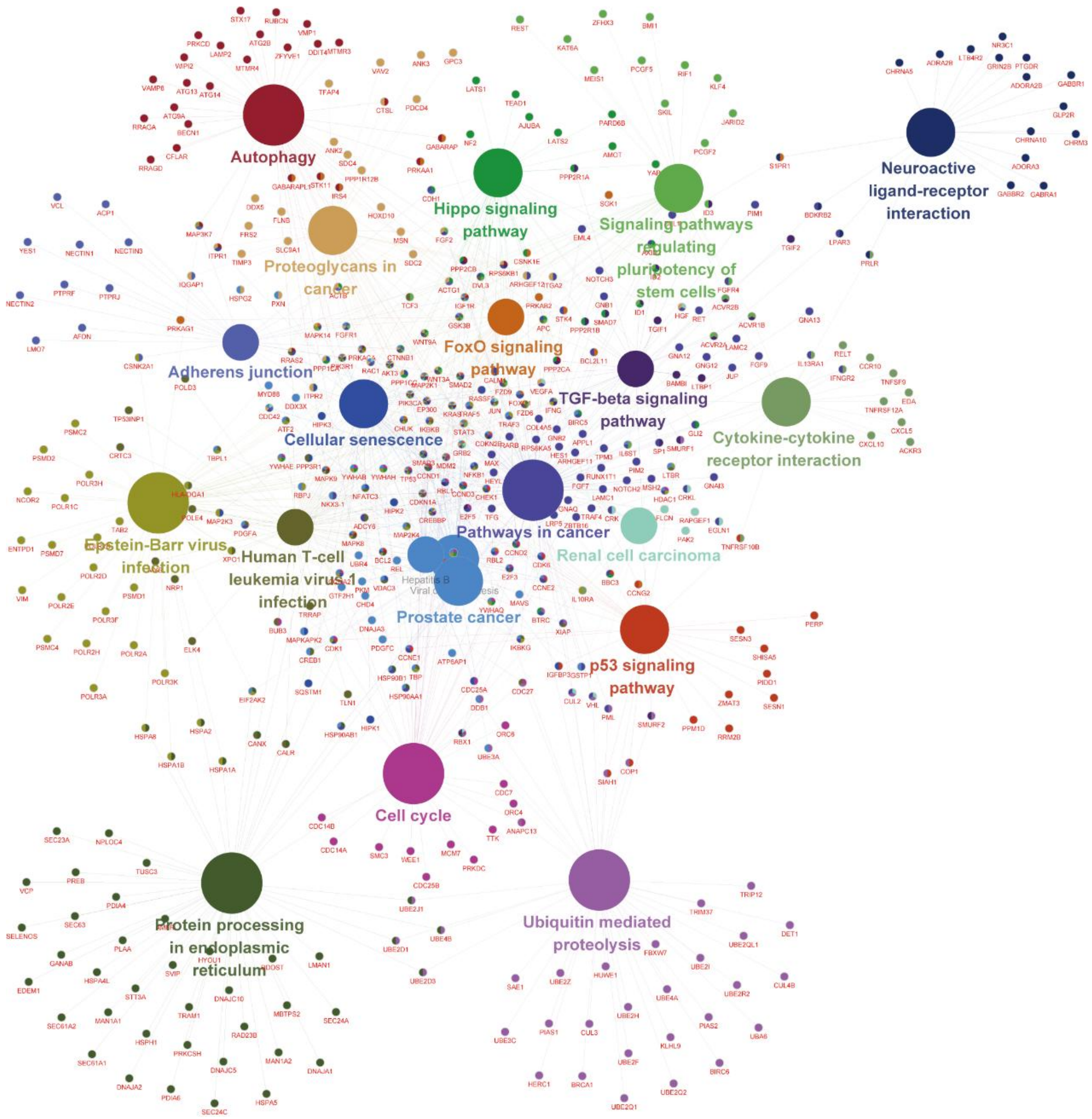

Supplementary Figure 9. KEGG pathway enrichment analysis of predicted miR-15a-5p target genes.

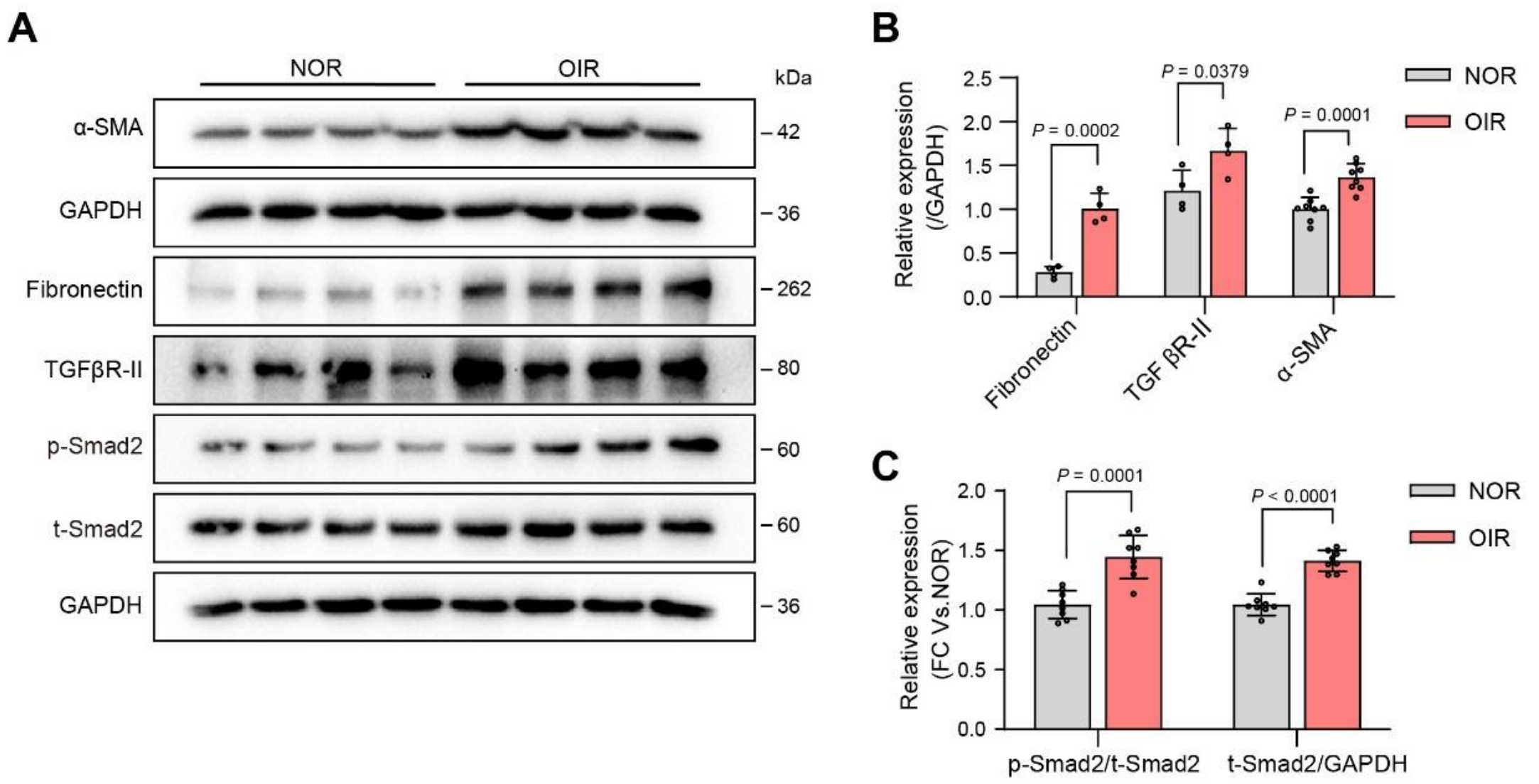

Supplementary Figure 10. Characterization of retinal fibrosis in OIR mice at P17. A. Representative western blot images showing the expression of fibronectin, TGFβR-II, α-SMA, p-Smad2, and t-Smad2 in the retinas of OIR mice at P17. **B.** Quantitative analysis of protein expression levels of fibrotic markers, fibronectin, TGFβR-II, and α-SMA (n = 4). **C.** Quantitative analysis of the p-Smad2/t-Smad2 and t-Smad2/GAPDH ratio (n = 4). Data in **B** and **C** are presented as mean ± SD and analyzed using an unpaired two-tailed Student's t-test.

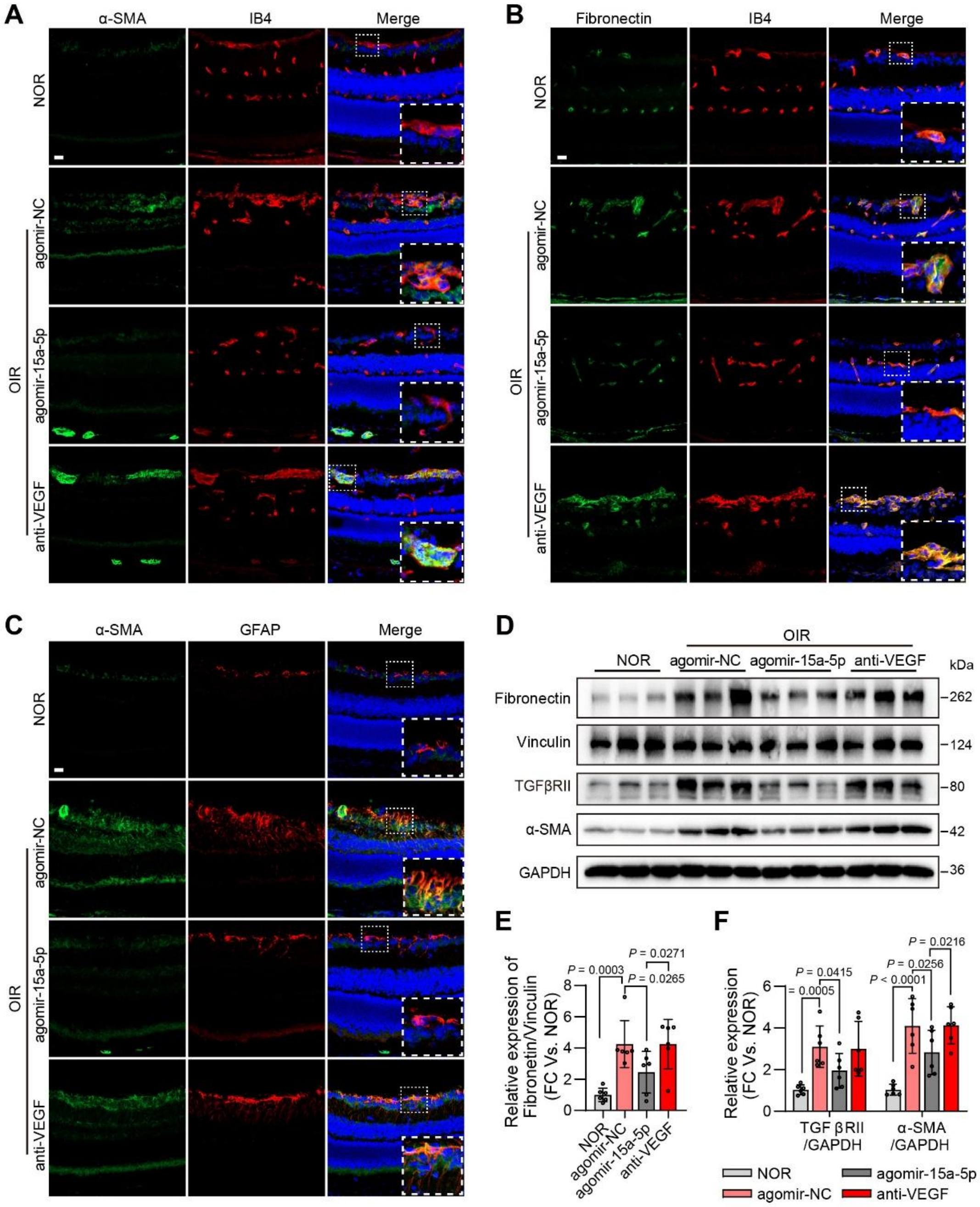

Supplementary Figure 11. miR-15a-5p suppresses retinal fibrosis in OIR mice at P17. A. Immunofluorescence staining for α-SMA (green) and blood vessels labeled with IB4 (red) in OIR retinas at P17. Nuclei were counterstained with DAPI (blue). **B.** Immunofluorescence staining for fibronectin (green) and IB4 (red). Nuclei were counterstained with DAPI (blue). **C.** Immunofluorescence staining for α-SMA (green) and GFAP (red). Nuclei were counterstained

with DAPI (blue). **D.** Representative western blot images showing the expression of fibronectin, α-SMA, and TGFβRII in OIR retinas at P17. **E-F.** Quantitative analysis of fibronectin (**E**), TGFβRII, and α-SMA (**F**) expression (n = 6). Data in **E** and **F** are presented as mean ± SD and analyzed using one-way ANOVA. Scale bar: 20 μm. The original Western blot images used for quantification in this study have been deposited in figshare and can be accessed at DOI: 10.6084/m9.figshare.30820226.

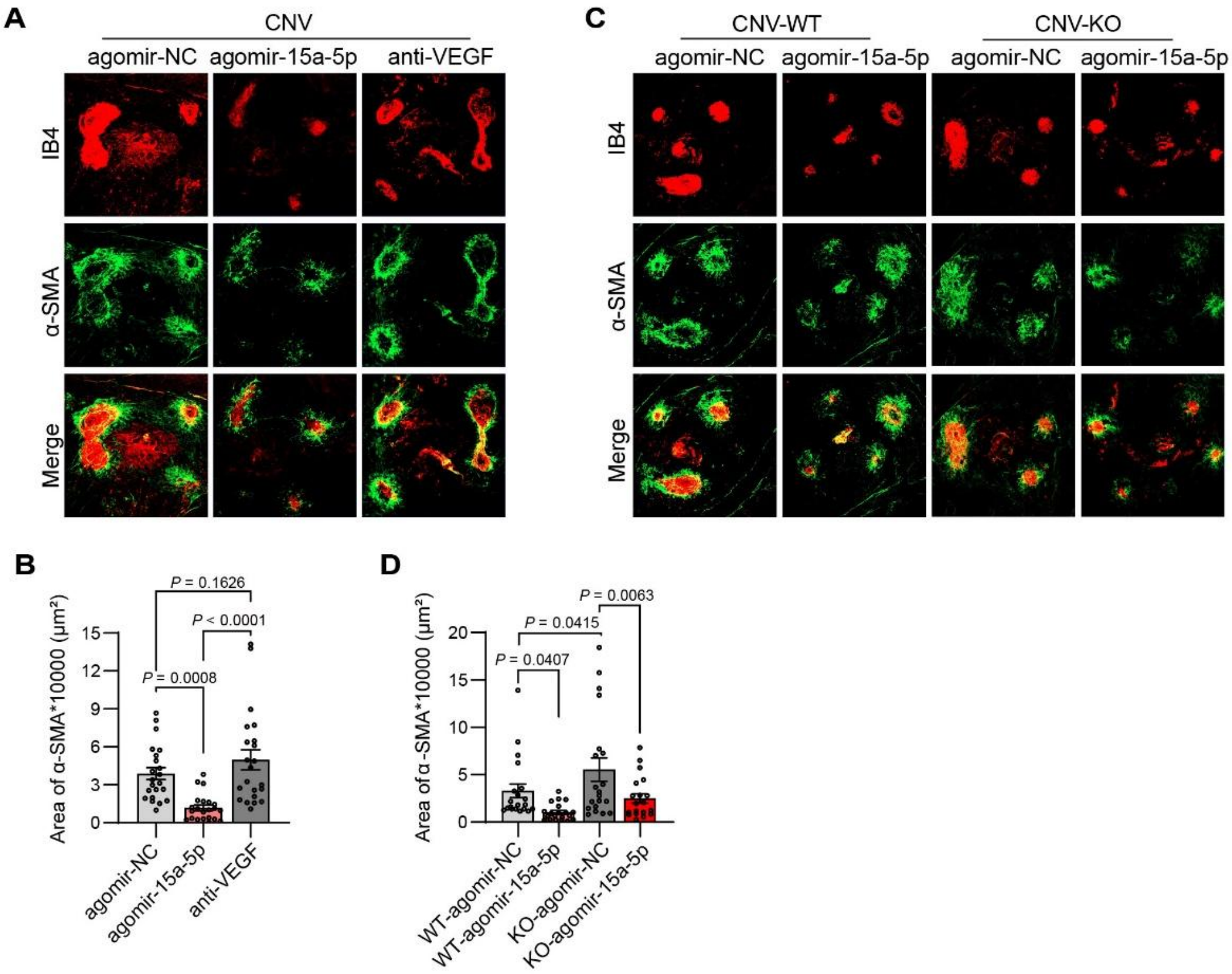

Supplementary Figure 12. miR-15a-5p deficiency promotes fibrosis in CNV lesions. A. Representative whole-mount immunofluorescence images of CNV lesions from WT mice after intravitreal injection of miR-15a-5p agomir or anti-VEGF drug at day 7. Blood vessels and myofibroblasts are labeled with IB4 (red) and α-SMA (green), respectively. **B.** Quantitative analysis of the α-SMA-positive fibrotic area in CNV lesions from WT mice following the indicated treatments (*n* = 22). **C**. Representative whole-mount immunofluorescence images of CNV lesions from miR-15a-5p KO and WT mice after intravitreal injection of miR-15a-5p agomir or anti-VEGF drug at day 7. Staining for IB4 (red) and α-SMA (green) is shown. **D.** Quantitative analysis of the α-SMA-positive fibrotic area in CNV lesions from miR-15a-5p KO and WT mice following the indicated treatments (*n* = 20). Data in **B** and **D** are presented as mean ± SD and analyzed using one-way ANOVA.

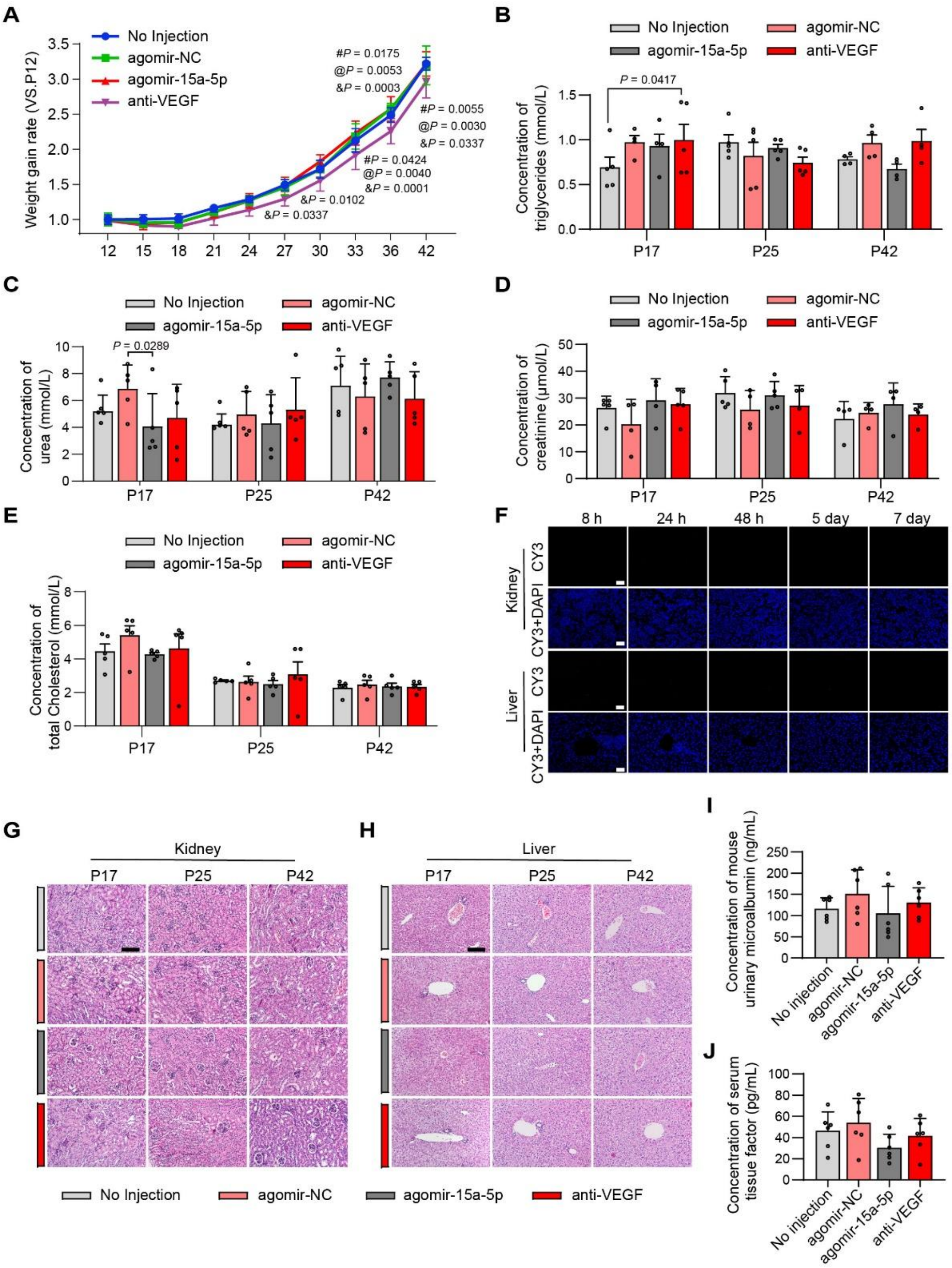

Supplementary Figure 13. Evaluation of the systemic safety of miR-15a-5p administration in normal mice. A. Body weight changes in non-injected (NI) and injected mice from P12 to P42 (n = 5–6). Symbols indicate comparisons between groups: #, NI vs. anti-VEGF; @, agomir-NC vs. anti-VEGF; &, agomir-15a-5p vs. anti-VEGF. **B–E.** Serum biochemical analyses showing triglyceride (**B**), urea (**C**), creatinine (**D**), and total cholesterol (**E**) levels in mice at P17,

P25, and P42 following intravitreal injection of different treatments (*n* = 4–5). **F.** Representative immunofluorescence images of kidney and liver sections from NOR mice at different time points after intravitreal injection of Cy3-labeled agomir-15a-5p. Nuclei were counterstained with DAPI (blue). Scale bar: 20 μm. **G–H.** Representative H&E staining images of kidney (**G**) and liver (**H**) sections from non-injected and injected mice at P17, P25, and P42. Scale bar: 20 μm. **I–J.** Quantification of urinary microalbumin (MAU) levels (**I**) and serum tissue factor (TF) levels (**J**) in mice at 5 days following intravitreal injection of the indicated treatments. Data in **A–E, I,** and **J** are presented as mean ± SD. Statistical significance was determined using one-way ANOVA.

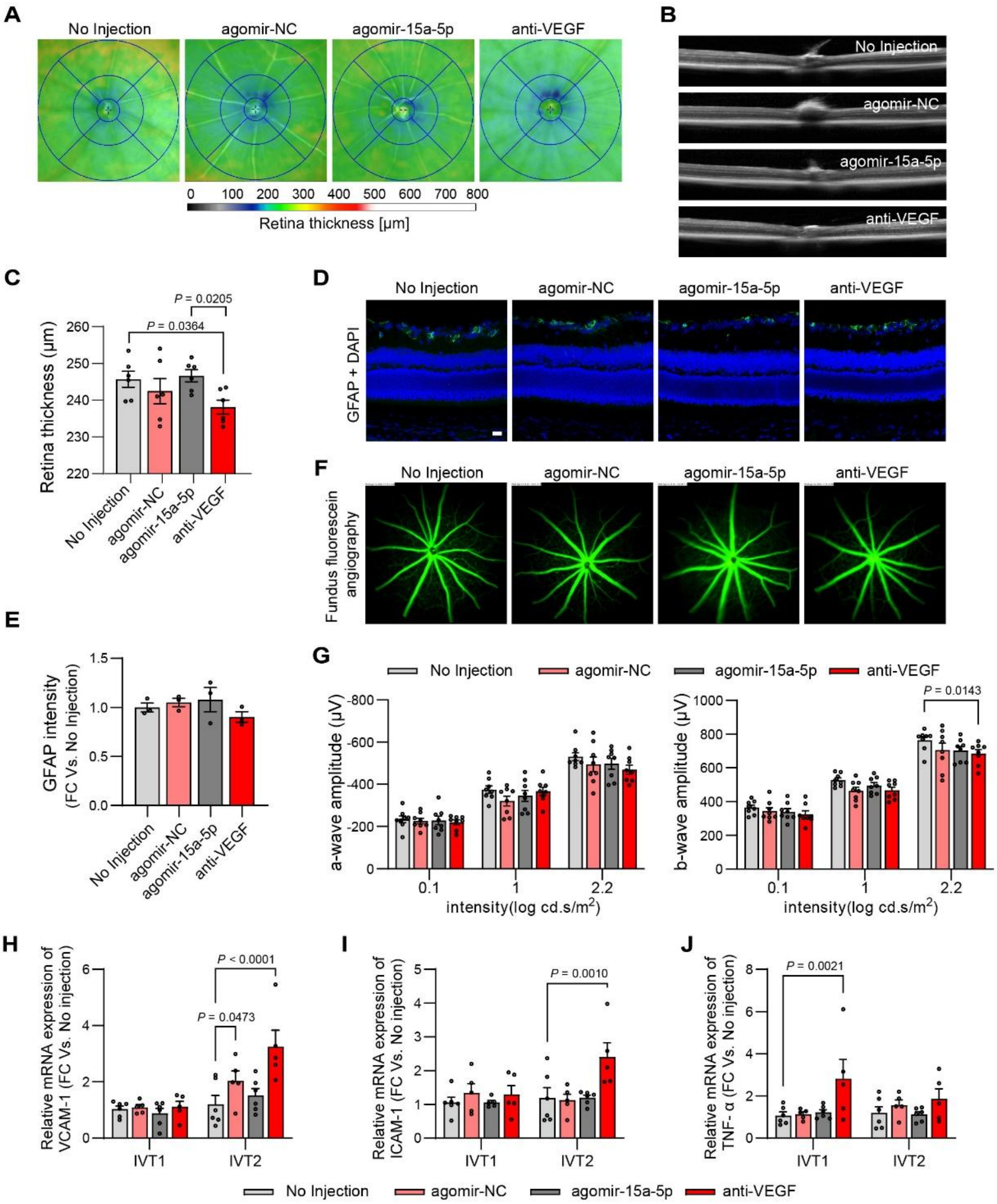

Supplementary Figure 14. Evaluation of ocular safety following intravitreal administration of miR-15a-5p and anti-VEGF drug in normal mice. A. Representative OCT images showing retinal thickness in mice at P42 after intravitreal injection at P12. **B.** Representative OCT cross-sections displaying retinal structure in each group at P42. **C.** Quantitative analysis of retinal thickness based on OCT measurements (n = 6). **D.** Representative retinal immunofluorescence images showing GFAP (green) expression in the

retina at P42. Nuclei were counterstained with DAPI (blue). Scale bar: 20 μm. **E.** Quantification of GFAP fluorescence intensity in the retina (*n* = 3). **F.** Representative fundus fluorescein angiography images of adult mice following intravitreal injection of the indicated therapeutic-dose treatments, showing intact retinal vasculature without detectable vascular leakage. **G**. Quantification of ERG a-wave and b-wave amplitudes in adult mice following intravitreal injection of the indicated therapeutic-dose treatments (*n* = 8). **H–J**. Quantitative PCR analysis of retinal inflammatory cytokine expression in adult mice following intravitreal injection of the indicated therapeutic-dose treatments (*n* = 5–6). IV1 represents retinas collected 5 days after a single intravitreal injection, whereas IV2 represents retinas collected 5 days after a second intravitreal injection administered 5 days following the first injection. Expression levels of VCAM-1 (**H**), ICAM-1 (**I**), and TNF-α (**J**) are shown. Data in **C, E, G, H, I,** and **J** are presented as mean ± SD and analyzed using one-way ANOVA.

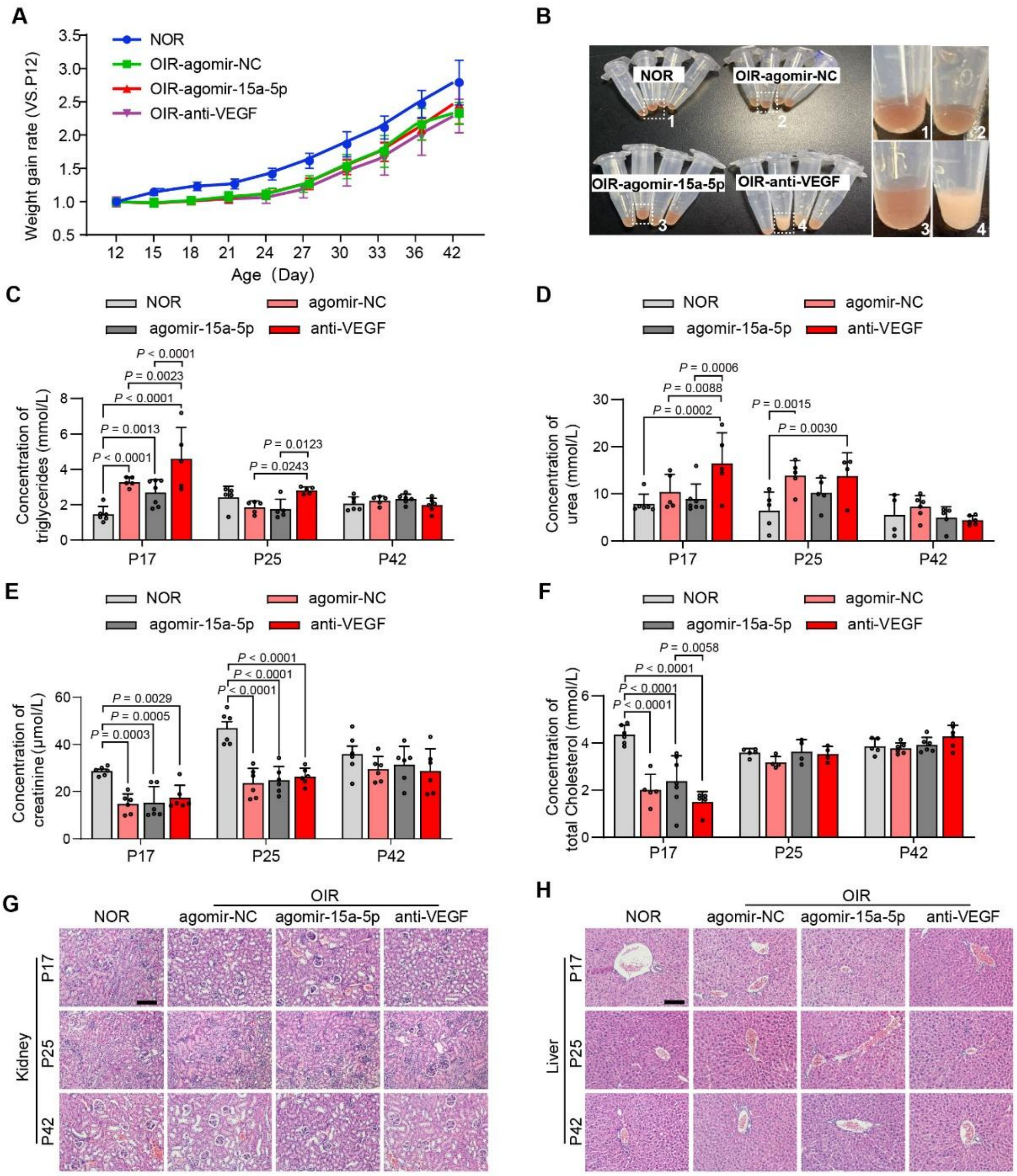

Supplementary Figure 15. Evaluation of systemic safety following intravitreal administration of miR-15a-5p in OIR mice. A. Body weight changes of NOR and OIR mice receiving different treatments from P12 to P42 (P12–P24, $n = 11$; P27–P42, $n = 7$). **B**. Representative images of serum samples collected from NOR and OIR mice at P42. **C–F**. Serum biochemical analyses showing triglycerides (**C**), urea (**D**), creatinine (**E**), and total cholesterol (**F**) levels in NOR and OIR mice at P17, P25, and P42 (n = 4-6). **G–H**. Representative H&E staining images of kidney (**G**) and liver

(**H**) sections from NOR and OIR mice at P17, P25, and P42. Scale bar: 20 μm. Data in **A, C, D, E,** and **F** are presented as mean ± SD. Statistical significance was determined using one-way ANOVA.

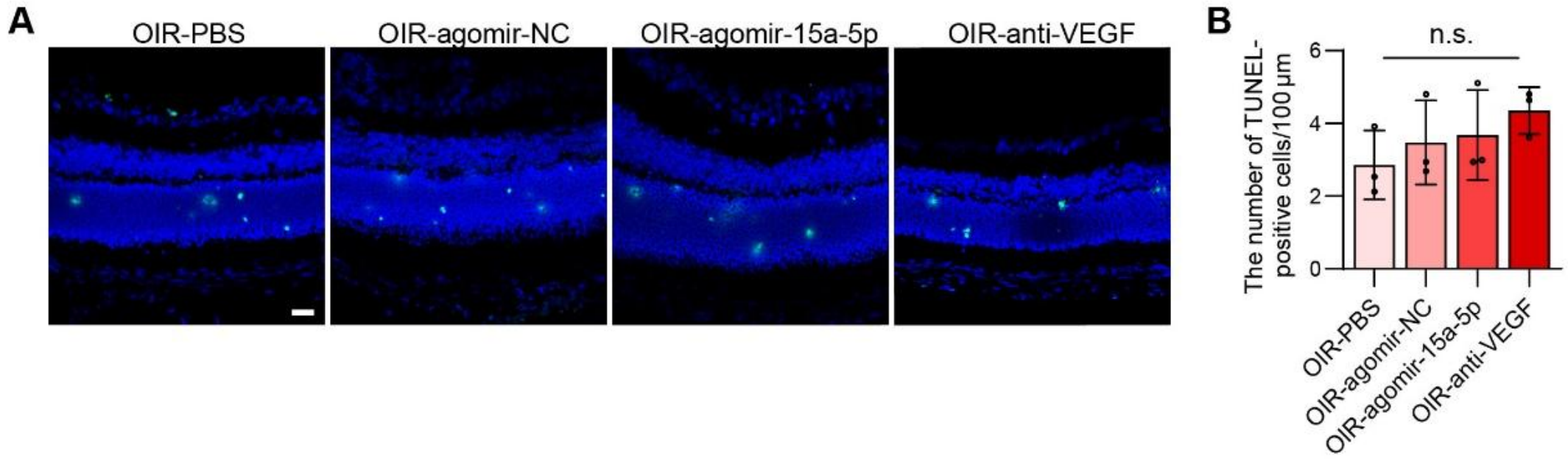

Supplementary Figure 16. Evaluation of retinal apoptosis following intravitreal administration in OIR mice. A. Representative images of retinal sections showing TUNEL staining (green) at P17 after intravitreal injection of agomir-NC, agomir-15a-5p, and anti-VEGF drug. Nuclei were counterstained with DAPI (blue). Scale bar: 20 μm. **B.** Quantitative analysis of TUNEL-positive nuclei per 100 μm of retinal length (*n* = 3). Data in **B** are presented as mean ± SD and analyzed using one-way ANOVA.

Supplementary Table 1. Baseline characteristics of the plasma cohort

Baseline demographic and clinical characteristics of participants included in the plasma cohort.

	NDM	NDR	NPDR	PDR	Statistics	*P* Value
Age (years)	62.59 ± 8.76	60.65 ± 10.99	60.81 ± 8.82	59.03 ± 9.27	F = 0.845	*P* = 0.471
Sex (male)	12	21	16	24	χ^2 = 7.793	*P* = 0.051
Duration of diabetes (years)	/	10.38 ± 3.98	13.31 ± 6.49	14.92 ± 7.9	H = 6.541	*P* = 0.038
HbA1c (%)	/	6.88 ± 0.79	7.00 ± 0.93	8.02 ± 1.79	F = 7.081	*P* = 0.001
Blood glucose, random (mmol/L)	5.50 (5.30–5.95)	7.65 (6.93–10.55)	8.15 (6.45–11.53)	8.25 (6.40–11.85)	H = 35.074	*P* < 0.001
Total cholesterol (mmol/L)	5.20 ± 1.04	4.84 ± 1.65	5.13 ± 1.02	4.67 ± 1.16	F = 1.473	*P* = 0.225
Triglycerides (mmol/L)	1.43 (1.12–1.93)	1.85 (1.20–2.70)	1.88 (1.17–2.97)	1.73 (1.03–2.50)	H = 4.334	*P* = 0.228
Blood urea nitrogen(mmol/L)	4.89 (4.13–5.51)	4.55 (3.81–5.52)	5.38 (4.39–7.04)	5.30 (4.23–6.91)	H = 7.458	*P* = 0.059
Serum creatinine (μmol/L)	61.10 (52.35–75.73)	64.05 (51.38–75.95)	69.25 (58.45–80.40)	69.50 (57.10–85.63)	H = 5.442	*P* = 0.142
n	34	34	36	38	None	None

Supplementary Table 2. Baseline characteristics of the vitreous cohort

Baseline demographic and clinical characteristics of participants included in the vitreous cohort.

	NDM	NDR	NPDR	PDR	Statistics	P Value
Age (years)	66.3 ± 7.0	68.0 ± 8.5	69.2 ± 12.2	61.8 ± 13.5	F = 1.034	0.388
Sex (male)	10	3	6	6	X^2 = 6.639	0.084
Duration of diabetes (years)	/	12.8 ± 9.4	16.2 ± 7.4	13.7 ± 6.9	F = 0.503	0.610
HbA1c (%)	/	6.70（5.95， 8.00）	7.90（6.65， 8.20）	7.80（6.60， 8.90）	H = 1.150	0.563
Blood glucose, random (mmol/L)	5.74 ± 0.79	6.93 ± 1.26	9.32 ± 4.86	7.81 ± 3.18	H = 9.979	0.019
Total cholesterol (mmol/L)	4.85 ± 1.01	4.22 ± 1.34	4.84 ± 1.16	4.31 ± 0.78	F = 1.952	0.137
Triglycerides (mmol/L)	1.44(1.09,1.62)	2.12(1.20,2.89)	1.27(0.99,1.86)	1.47(1.08,2.02)	H = 1.918	0.590
Blood urea nitrogen (mmol/L)	4.55 ± 1.13	5.41 ± 1.90	6.11 ± 2.18	5.58 ± 1.73	F = 1.818	0.159
Serum creatinine (µmol/L)	70.01 ± 12.81	77.29 ± 24.00	85.33 ± 28.22	67.63 ± 15.13	F = 1.606	0.204
IOP (mmHg)	13.46 ± 3.20	14.48 ± 2.25	15.28 ± 3.94	13.81 ± 3.25	F = 0.640	0.594
n	12	10	10	12	/	/

Supplementary Table 3. Baseline characteristics of the aqueous cohort

Baseline demographic and clinical characteristics of participants included in the aqueous cohort.

	Control	AMD	Statistics	P Value
Age (years)	69.0 ± 1.6	68.4 ± 1.9	t = 0.227	0.822
Sex (male)	7	5	X^2 = 0.467	0.495
IOP (mmHg)	13.88 ± 0.55	13.89 ± 0.56	t = 0.012	0.990
Blood glucose, random (mmol/L)	5.7（4.9, 6.7）	7.0 (5.2, 8.6)	U = 170.500	0.208
Total cholesterol (mmol/L)	5.72 ± 0.26	5.36 ± 0.12	t = 1.248	0.219
Triglycerides (mmol/L)	1.72 (1.24, 2.61)	1.53 (1.19, 2.48)	U = 401.500	0.489
Blood urea nitrogen (mmol/L)	5.61 ± 0.33	5.00 ± 0.23	t = 1.549	0.129
Serum creatinine (μmol/L)	73.09 ± 3.12	69.20 ± 3.49	t = 0.832	0.411
n	21	21	/	/

Supplementary Table 4. Key resources

A list of the key reagents, materials, and resources used in this study, including the corresponding manufacturers and catalog numbers.

REAGENT or RESOURCE	SOURCE	IDENTIFIER
Antibody		
Rabbit polyclonal anti-GFAP	Abcam	Cat# ab7260; RRID: AB_305808
Rabbit polyclonal anti-GAPDH	Proteintech	Cat# 10494-1-AP; RRID: AB_2263076
Rabbit monoclonal anti-ICAM-1	Abcam	Cat# ab179707; RRID: AB_2814769
Rabbit polyclonal anti-VEGF	Abcam	Cat# ab46154; RRID: AB_2212642
Rabbit monoclonal anti-t-ERK	Cell Signaling Technology	Cat# 4695; RRID: AB_390779
Rabbit monoclonal anti-p-ERK	Cell Signaling Technology	Cat# 9101; RRID: AB_331646
Rabbit monoclonal anti-p-Smad2	Cell Signaling Technology	Cat# 18338; RRID: AB_2798798
Rabbit monoclonal anti-t-Smad2	Cell Signaling Technology	Cat# 5339; RRID: AB_10626777
Rabbit monoclonal anti-Vimentin	Abcam	Cat# ab92547; RRID: AB_10562134
Mouse monoclonal anti-CD31	Abcam	Cat# ab9498; RRID: AB_307284
Rabbit monoclonal anti-α-SMA	Abcam	Cat# ab124964; RRID: AB_11129103
Rabbit monoclonal anti-Fibronectin	Abcam	Cat# ab45688; RRID: AB_732380
Rabbit monoclonal anti-TGFβR-II	Abcam	Cat# ab259360; RRID: AB_3668832
Rabbit monoclonal anti-Vinculin	Abcam	Cat# ab129002; RRID: AB_11144129
Rabbit polyclonal anti-CD63	Abcam	Cat# ab216130; RRID: AB_3076642
Rabbit monoclonal anti-ApoA1	Proteintech	Cat# 83504-1-RR; RRID: AB_3671129
Rabbit polyclonal anti-TSG101	Proteintech	Cat# 28283-1-AP; RRID: AB_2881104
Reagent		
Lectin GS-IB4	Invitrogen	Cat# I21413; RRID: AB_2313921
lipo 3000	Thermofisher	Cat# L3000001
VEGF	MCE	HY-P7110
TGF-β1	PeproTech	Cat# AF-100-21C-50
Kit		
TUNEL	Roche	11684817910
ELISA (TNF-α)	Abcam	ab181421
ELISA (VEGF)	Abcam	ab222510
Urea colorimetric test kit	Elabscience	Cat#E-BC-K183-M-96T
Triglyceride colorimetric test kit	Elabscience	Cat#E-BC-K261-M-96T
Creatinine colorimetric test kit	Elabscience	Cat#E-BC-K188-M-96T
Total cholesterol colorimetric test kit	Elabscience	Cat#E-BC-K109-M-96T
Animal		
C57BL/6JGpt mice	GemPharmatech	Strain NO.N000013; RRID: IMSR_GPT:N000013
C57BL/6JGpt-Mir15aem23Cd777Mir16-1em1Cd/Gpt	GemPharmatech	Strain NO. T050727<br>RRID: IMSR_GPT:T050727
